# Biogenic iron oxide nanoparticles enhance callogenesis and regeneration pattern of recalcitrant *Cicer arietinum* L.

**DOI:** 10.1371/journal.pone.0242829

**Published:** 2020-12-01

**Authors:** Samra Irum, Nyla Jabeen, Khawaja Shafique Ahmad, Saima Shafique, Talha Farooq Khan, Hina Gul, Sadaf Anwaar, Nuzhat Imam Shah, Ansar Mehmood, Syed Zaheer Hussain

**Affiliations:** 1 Applied Biotechnology and Genetic Engineering Lab, Department of Biological Sciences, International Islamic University, Islamabad, Pakistan; 2 Department of Botany, University of Poonch, Rawalakot (UPR), Rawalakot, Azad Jammu and Kashmir, Pakistan; 3 Department of Materials Science & Engineering, Institute of Space Technology Islamabad, Islamabad, Pakistan; 4 University Institute of Biochemistry and Biotechnology, PMAS Arid Agriculture University, Rawalpindi, Pakistan; 5 Department of Microbiology, Hazara University, Mansehra, Pakistan; 6 Department of Biological Sciences, Quaid-i-Azam University, Islamabad, Pakistan; Government College University Faisalabad, PAKISTAN

## Abstract

This study is the first report on the biosynthesized iron oxide nanoparticles (IONPs) which mediate in-vitro callus induction and shoot regeneration in economically important recalcitrant chickpea crop (*Cicer arietinum* L.). Here, we used leaf extract of *Cymbopogon jwarancusa* for the synthesis of IONPs in order to achieve a better biocompatibility. The bioactive compounds in *C*. *jwarancusa* leaf extract served as both reducing and capping agents in the fabrication process of IONPs. Field emission scanning electron microscopy (FE-SEM) revealed rods like surface morphology of IONPs with an average diameter of 50±0.2 nm. Energy-dispersive X-ray spectroscopy (EDS) depicted formation of pure IONPs with 69.84% Fe and 30.16% O_2_. X-ray diffractometry (XRD) and attenuated total reflectance-fourier transform infrared (ATR-FTIR) validate the crystalline structure, chemical analysis detect the presence of various biomolecular fingerprints in the as synthesized IONPs. UV-visible absorption spectroscopy depicts activity of IONPs under visible light. Thermo-gravimetric analysis (TGA) displayed thermal loss of organic capping around 500°C and confirmed their stabilization. The biosynthesized IONPs revealed promising results in callus induction, shoot regeneration and root induction of chickpea plants. Both chickpea varieties Punjab-Noor 09 and Bittle-98 explants, Embryo axes (EA) and Embryo axes plus adjacent part of cotyledon (EXC) demonstrated dose-dependent response. Among all explants, EXC of Punjab-Noor variety showed the highest callogenesis (96%) and shoot regeneration frequency (88%), while root induction frequency was also increased to 83%. Iron content was quantified in regenerated chickpea varieties through inductively coupled plasma-optical emission spectrometry. The quantity of iron is significantly increased in Punjab-Noor regenerated plants (4.88 mg/g) as compare to control treated plants (2.42 mg/g). We found that IONPs enhance chickpea growth pattern and keep regenerated plantlets infection free by providing an optimum environment for rapid growth and development. Thus, IONPs synthesized through green process can be utilized in tissue culture studies in other important recalcitrant legumes crops.

## Introduction

Chickpea (*Cicer arietinum* L.) is one of the most important legume crop in the world with an annual yield of 11.6 million tons [1]. Chickpeas are rich source of proteins, carbohydrates, vitamins and minerals, and they provide diversified human health benefits and nutritional values [2]. Different chickpea derivatives such as culled chickpea, low-grade pea, chickpea pod husks, and hay are widely used as protein-rich feed in animal diet and chickpea straw as alternative forage for ruminants [3]. The crop is used in agriculture system for sustainable production because it provides biologically fix nitrogen, and maintains soil productivity [4]. The demand of high quality chickpeas is increasing steadily with increasing world population [5]. While, global chickpea yield does not meet the present demand due to various biotic and abiotic factors [6]. Fungal diseases, mainly Ascochyta blight and Fusarium wilt are the major biotic constraints involved in significant yield loss of chickpea crop worldwide [7]. To overcome these production restraints, it is imperative to use advanced biotechnological approach for the improvement of legumes and to understand the genetics of multifaceted traits [8]. Plant tissue culture technique is used to screen plantlets and provide opportunity to study the different aspects of plant growth and development. Additionally, this technique has become vital for the selection of plants resistance to several abiotic and biotic stresses [9]. The biggest advantage of in-vitro micro-propagation of crops is the production of more number of plants in a very short time under controlled environmental conditions [10]. Nanotechnology is an emerging field that has novel applications in agriculture and plant biotechnology [11]. Nanoparticles (NPs) can be synthesized from different organic and inorganic materials. However, green synthesis of metallic NPs have received much attention due to their wide applications in various fields of science and technology including electronics to structural engineering and agriculture to medicine [12]. Plant extract mediated synthesis has advantage over chemical synthesis of nanoparticles because of low cost, safe and ease large scale synthesis of NPs [13]. Green synthesized nanoparticles have small size (less than 100 nm), high surface area, and eco-friendly nature [14]. Plant extracts are comprised of natural compounds such as flavonoids, saponins, terpenes, phenolics, tannins, amino acids, inositol, vitamins and resins [15]. These bioactive compounds in plant extract function as natural capping agent, reducing and stabilizing agents, and results in formation of capped NPs [16]. Green synthesized nanomaterial’s enhance the productivity of crops, accelerate plant germination and regeneration capacity, and develop crop resistance against various biotic and abiotic stresses. It is, therefore, vital to use micronutrients in the form of nanoparticles to increase the crop productivity [17]. Iron oxide nanoparticles (IONPs) are one of the most important oxides and possess a wide range of applications in agriculture, cosmetics, biomedicine, diagnostics, material engineering, and bioremediation [18, 19]. IONPs have potential applications as crop fertilizers due to their environmental benign nature, magnetically sensitivity, readily availability, redox activity and chlorophyll biosynthesis [20, 21]. Iron oxide NPs exist in a diversified polymorphic form including maghemite (γ-Fe_2_O_3_), goethite FeO(OH), and hematite (α-Fe_2_O_3_) [22]. Among all of them, hematite (α-Fe_2_O_3_) is thermodynamically and chemically more stable form of iron-oxide nanoparticles because of its rhombohedral geometry and centered hexagonal structure with dense-packed O_2_ lattice [23]. Iron is an important micronutrient, which involves chlorophyll formation and cellular reactions in plants. Iron plays a significant role in plant growth and metabolism, phytohormonal regulation, mainly auxins, carbohydrate metabolism, protein synthesis, and stress-related response [24]. In plants, iron uptake is highly regulated through apoplastic pathway to supply sufficient amount for optimal growth [25]. Iron oxide nanoparticles obtained from green synthesis method are nontoxic to humans and are more biocompatible when compared to their chemically synthesized counterparts [26]. IONPs have significantly enhanced growth in the strawberry, corn, tomato, rapeseed, and sorghum [27–29].

To the best of our knowledge no research is available about impact of green synthesized IONPs on tissue culture studies of recalcitrant chickpea cultivars. In this research, we present a novel green fabrication procedure for the synthesis of iron oxide nanoparticles using *C*. *jwarancusa* aqueous leaves extract. Further, biosynthesized IONPs are evaluated to boost callogenesis, shoot regeneration dynamics, shoot elongation and root induction in recalcitrant chickpea varieties (Punjab-Noor 09 and Bittle-98). It is believed that these findings will help researchers to mitigate the scarcity of food supply and related malnutrition by improving agricultural productivity of economically important legume crops.

## Materials and methods

### Green synthesis of iron oxide nanoparticles

*Cymbopogon jwarancusa* leaves extract was used for the synthesis of IONPs ascribing its pharmacological potential. Briefly, fresh plant sample of *C*. *jwarancusa* grass were collected from the northern region of the Nara desert of Pakistan and authenticated by an expert taxonomist. Firstly, fresh leaves were washed three times with double distilled water (d.dH_2_O) to remove dust particles and other contaminated organic materials. To avoid dissociation of bioactive compounds, collected plant leaves were shade dried at room temperature. Afterwards, dried leaves were crushed into a fine powder using Wiley Mill grinder (Thomas Scientific ED-5). For extraction purpose, 30 g of fine leaf powder was dissolved into 300 ml of d.dH_2_O (Mili-Q Merck^™^) in an Erlenmeyer flask and kept on boiling for 30 min at 100°C. In the next step, flask was placed on orbital shaking incubator set to 37°C at 100 rpm for overnight to obtain phytochemical enrich extract. Subsequently, leaves extract was vacuum filtered through Whatman^™^ 41 filter paper (GE Health care, 20 μm) and filtrate extract was stored at 4°C in the refrigerator for further use [30].

**Fig 1:**
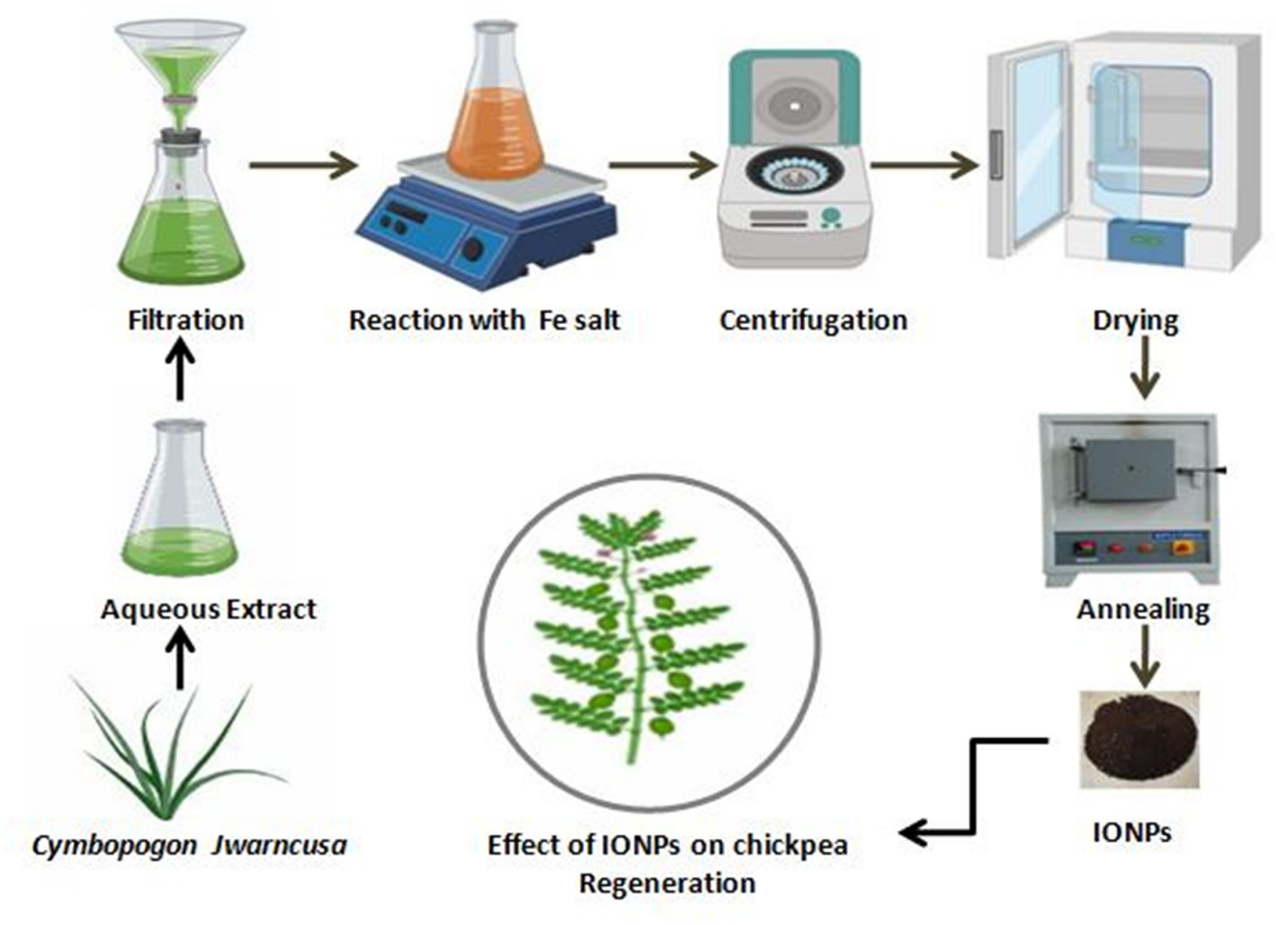
Stepwise schematic protocol for green synthesized IONPs using leaves extract of *C*. *jwarancusa*.

For green synthesis of IONPs, Iron (III) nitrate nonhydrate (Fe(NO_3_)_3_^.^9H_2_O), (Sigma Aldrich) was used as a precursor of iron salt (Fe^+3^). Briefly, 12.12 g salt was added to 300 ml of *C*. *jwarancusa* leaves extract (0.5M, pH 5.7). The reaction solution was stirred on hot plate magnetic stirrer (IKA^™^) heated to 70°C at 1200 rpm for 2 h. Following, reaction mixture was centrifuged at 10,000 rpm (GR-BioTek) for 15 min till brownish-red color pellet of IONPs was collected. The pellet was then washed thrice with d.dH_2_O to remove impurities, thereafter dehydrated in hot air drying oven (Memmert) at 65°C for 6 h (Fig 1). In order to achieve better crystalline structures, IONPs were calcinated inside Gallenkamp Muffle-furnace for 2 h at 500°C [31].

### Characterization of IONPs

Field emission scanning electron microscopy (FESEM, TESCAN, MIRA3 Czech Republic) was used for surface morphological characterization at 20 kV with a counting rate of 2838 and it was equipped with energy-dispersive X-ray spectroscopy (EDS, Oxford) for chemical compositional analysis of green synthesized IONPs. The FE-SEM micrographs of IONPs were taken at 10kX, 25kX, 50kX and, 100kX resolutions. Particle size analysis was performed using particle size analyzer (PSA, MALVERN master sizer hydro 3000, UK). Crystallographic parameters and phase purity of IONPs were characterized using X-ray powder diffraction (XRD) spectroscopy (XRD; Explorer, G.N.R. Analytical Instruments Group, Italy). The scanning rate of 2 μ angles/min with an operating voltage of 40 kV and 30 mA current was supplied in an ambient environment. The diffraction range of 2θ was set from 20° to 80° and average crystallite size (D) was evaluated using Debye Scherrer’s [D = 0.9λ/βcos] equation.

ATR-Fourier transform infrared spectroscopy (ATR-FT-IR, NICOLET iS10, Thermo Scientific, USA) was performed within the spectral range of 500–4000 cm^-1^ and obtained peaks were compared with the standard database to detect the chemical fingerprints present in the IONPs. The light absorption characteristics of the biosynthesized IONPs were evaluated through UV-visible spectroscopy in the range between 200–800 nm (Shimadzu spectrophotometer UV-1800, Kyoto, Japan) under ambient conditions. For this, 1 mg of IONPs was dissolved in deionized water and suspension was sonicated for 25 min. Subsequently, the sample was placed in the UV-visible spectrophotometer. Moreover, thermo gravimetric analysis (TGA, 1 STAR^e^ system, METTLER TOLEDO, USA) was carried to analyze the progressive weight loss, thermal stability, and bimolecular capping around green fabricated IONPs. The temperature range in TGA was set between 25–1000°C with an increment of 10°C per minute.

### Collection of plant material and explants preparation

Seeds of two Pakistani chickpea cultivars namely, Punjab-Noor 09 (Kabuli type) and Bittle-98 (Desi variety) were obtained from National Agriculture and Research Center (NARC), Islamabad Pakistan. Seeds were surface sterilized with 70% ethanol for 3 min, followed by 40% commercial Clorox treatment for 20 min. Subsequently, seeds were rinsed three times with sterile distilled water (Mili-Q, Merck^™^) and sterilized chickpea seeds were soaked in sterile distilled water for 16 h under aseptic conditions. Next day, the soaked chickpea seeds were decoated and two sets of explants were designed. One set of embryo axes (EA) with removed root apex and the second set with embryo axes (EXC) plus adjacent part of the cotyledon with removed root apex was used as explants.

### Media preparation

Murashige and Skoog medium (MS) 4.43 g/L, with pH 5.8, vitamin B12 (I00X) along 3% (w/v) of sucrose as carbon source was thoroughly mixed. About 3% Gellan-gum (PhytoTech Labs) was added to the media as a solidifying agent and autoclaved at 121°C for 20 min. Different concentrations of IONPs (1, 5, 10, 15, 20 mg/L) were added to autoclaved MS media. To avoid agglutination of nanoparticles at the bottom of the flask, the media was allowed to cool up to 45°C. In the next step, media flask was kept at room temperature till solidification and all the work was carried under sterilized conditions.

### Influence of IONPs on callus induction

For callus induction, MS media with 2,4-D (1.5 mg/L of 2,4-Dichlorophenoxyacetic acid) was used and supplemented with various concentrations of IONPs (1, 5, 10, 15, 20 mg/L) and without IONPs as a control. The culture tubes were set aside in dark and white fluorescent light (40-50/μmoL^.^m^2^/s, 16/8 h photoperiod) conditions at 25±2°C for 14 days. After a cultivation period of 13–16 days, embryogenic callus induced from explants were achieved [32]. Callus induction frequency was calculated using the following formula:

$$Callus\;Induction\;frequency\;(\%)=\frac{No.of\;Calli\;produced\;by\;explant}{No.of\;explant\;inoculated}\times 100$$ (1)

### Influence of IONPs on organogenesis

Both varieties of explants (EA+EXC) were investigated on shoot regeneration media (SRM) containing different concentrations of cytokinin (BAP) and kinetin (Kn). SRMI (MS+ 0.5mg/L BAP), SRMII (MS+ 1 mg/L BAP+ 0.5 mg/L Kn) supplemented with different concentrations of IONPs (1–20 mg/L) and without IONPs as control. The regeneration frequency was observed as the percentage of chickpea explants respond to the development of shoot after 14 days [32]. The regeneration frequency was calculated using the formula given below:

$$Regeneration\;Frequency\;(\%)=\frac{No.ofexpalnts\;regenerated\;into\;plantlets}{No.of\;explants\;inoculatedfor\;regeneration}\times 100$$ (2)

### Influence of IONPs on shoot elongation

Shoot elongation is a necessary step in organogenesis as it gives strength to the plant stem. Shoots growing on shoot regeneration media were carefully separated from an intact basal part. Healthy shoots were then selected and shifted to shoot elongation media (SE). Different concentrations of IONPs (1–20 mg/L) with a combination of Thiodiazuron (TDZ, 1 mg/L) were used for shoot elongation. The average length of shoot induced on SE was determined after 2 weeks post-transfer to observe the best shoot elongation. The cultures were kept at 25±2°C under white fluorescent light for 16 h photoperiod.

### Influence of IONPs on root induction

To assess the effect of IONPs on rooting, healthy shoots were transferred to root induction media (RIM). The regenerated shoots were cultured on MS media supplemented with different concentrations of IONPs (1–20 mg/L) along with indole 3-butyric acid (IBA, 1.5 mg/L). Growth conditions were same as 25±2°C for 16 h photoperiod under white fluorescent light.

### Determination of iron content in regenerated chickpeas

The regenerated healthy fresh shoots were carefully harvested, washed with distilled water, dehydrated in oven at 60°C for^˷^ 18 h, and then homogenized. These homogenized shoots were digested in the acid mixture having concentrated H_2_SO_4_, HNO_3,_ and HCIO_4_ (60%) acids (1:3:1) for 24 h in Single Reaction Chamber Microwave digestion system (MILESTONE, LabTech, Italy). Later, final volume of 10 ml was made with distilled H_2_O. The amount of iron present in sample solution was determined via inductively coupled plasma-optical emission spectrometry (OES-ICAP6500, Thermo Scientific) and is expressed as mg/g (dry weight) of plant tissue [33].

### Statistical analysis

All the data were examined statistically using OriginPro^™^-8.5 software. All the experiments were repeated thrice (n = 3) and each replication has 20 observation.

## Results and discussion

### Characterization of IONPs

A rapid, simple and cheap method for the green synthesis of IONPs has been efficaciously demonstrated for first time using leaf extracts of *C*. *jwarancusa*. Biogenic synthesis is effective because it is relatively safer when compare to chemical and physical methods and hence can efficiently use for biological applications.

**Fig 2:**
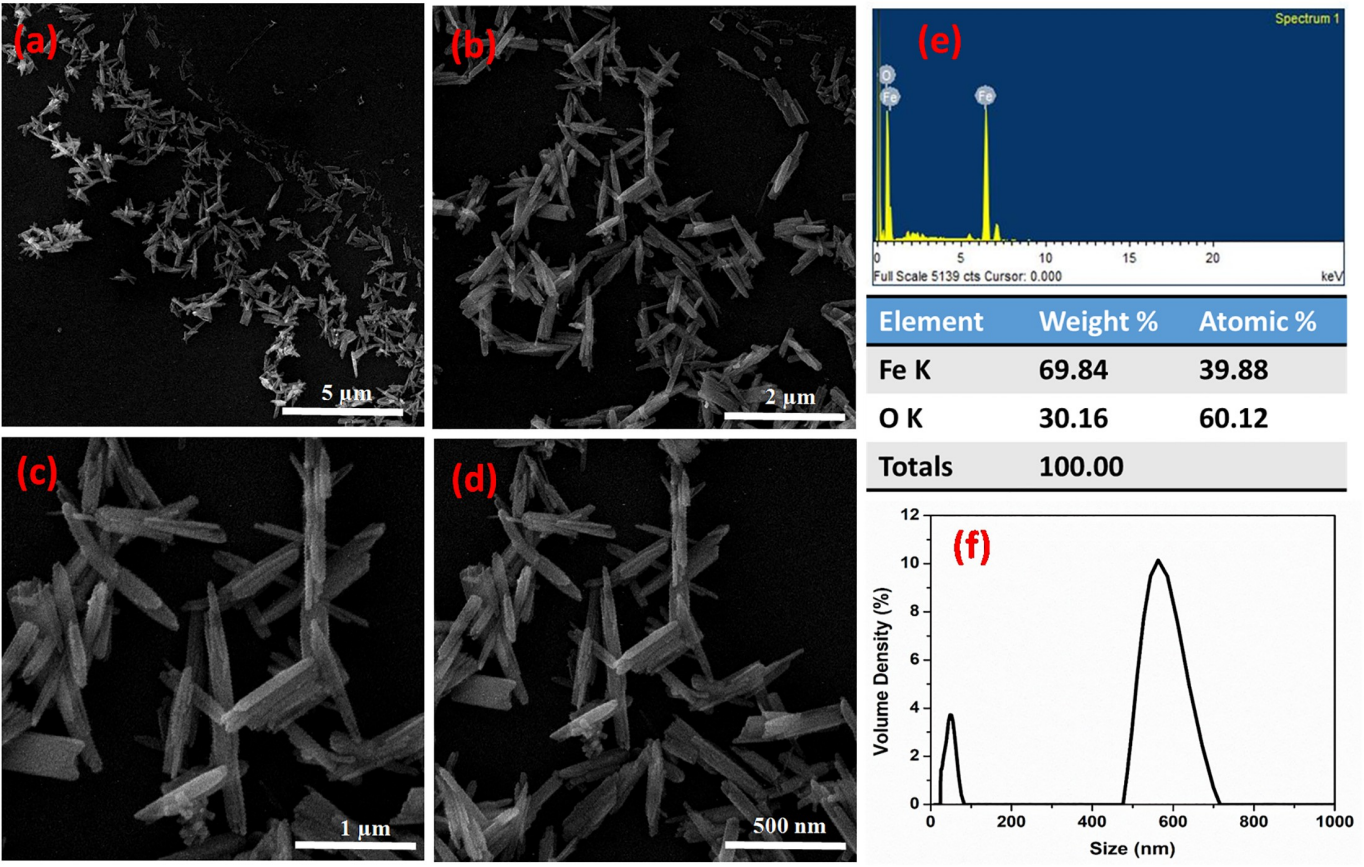
Field emission scanning electron microscope (FE-SEM) micrographs of IONPs showing nanorods at a resolution of (a) 10 KX (b) 25 KX (c) 50 KX (d) 100 KX (e) Particle size distribution of IONPs.

The morphology of green synthesized IONPs were examined through a Field emission scanning electron microscope (FE-SEM) while its chemical composition was confirmed through energy-dispersive X-ray spectroscopy (EDS) as shown in Fig 2. IONPs prepared through green method have highly crystalline needle-like shape and rod-like morphology, henceforth, these NPs may also be referred to as hematite Fe_2_O_3_ nanorods. Fig 2a–2d) presents SEM micrographs of Fe_2_O_3_ nanorods at an increasing magnification of 5μm, 2 μm, 1 μm, and 500 nm, respectively, where individual nanorods were observed at higher magnification. The diameter of these nanorods ranged between 38–79 nm. A high concentration of Fe_2_O_3_ nanorods was^˷^50 nm in size.

EDS spectrum in Fig 2e depicts that Fe_2_O_3_ nanorods are of high purity since only two peaks of iron and oxygen were observed. The major peak indicates the presence of Fe (69.84 wt. %) while the other refers to the remaining oxygen content (30.16 wt. %). The current findings are supported by previous reports; where Al-Ruqeishi et al. [34] and Aisida et al. [35] reported similar green synthesized iron oxide nanorods like morphology. In our findings, Fe_2_O_3_ nanorods-like morphology of green synthesized IONPs indicated successful capping of natural compounds from *C*. *jwarancusa* extracts. The average size of IONPs was measured using particle size analyzer (PSA). As shown in Fig 2f, we observed two different peaks, one at an average particle size of about 50nm while the other at around 560 nm. By interconnecting the data obtained through SEM with particle size analysis, we can suggest that the smaller peak gives a measure of the average diameter of the Fe_2_O_3_ nanorods, while the larger peak resulted from the measurement along the length of the Fe_2_O_3_ nanorods.

**Fig 3:**
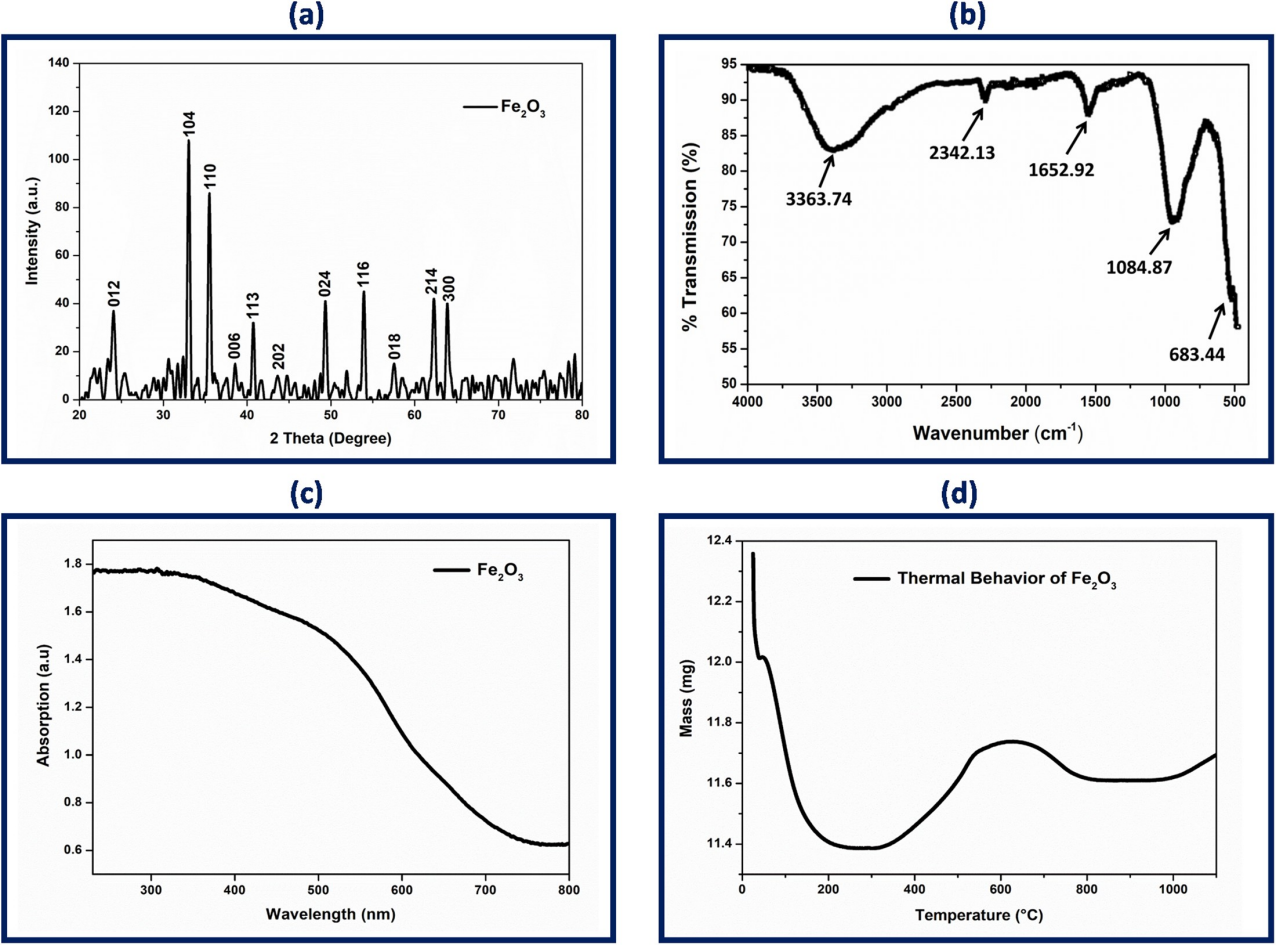
(a) X-ray powder diffraction (XRD) patterns of biosynthesized α-Fe_2_O_3_ demonstrating Braggs diffraction peak associated to Fe crystallites planes, (b) ATR-FTIR spectrum of green synthesized IONPs showing bond vibrations coming from surface attached bioactive compounds and Fe-O, (c) UV-Vis spectrum of IONPs, and (d) Thermogravimetric analysis (TGA) of the prepared IONPs.

The crystalline nature of green fabricated IONPs was investigated through X-ray diffraction. The XRD pattern clearly indicated eleven distinct peaks indexed to (012), (104), (110), (006), (113), (202), (024), (116), (018), (214), and (300), corresponding to the 2θ values of 24.1, 33.2, 35.7, 38.9, 40.9, 49.4, 54.1, 57.6, 62.6, and 64.0°, respectively (Fig 3a). The comparatively higher (104) peak reveals crystal planes of rhombohedral crystal structure and suggests ideal orientation of the hematite crystallites. All noted peak intensity scans were indexed to the rhombohedral α-Fe_2_O_3_, having lattice parameters a = 5.034 Å, b = 5.034 Å and c = 13.746 Å, in accordance with JCPDS card no. 79–1741. The same crystallographic pattern of IONPs was also observed by Jacob et al. [36] and Trpkov et al. [37]. The average crystallite size of α-Fe_2_O_3_ was found to be 50±0.2 nm using debye-scherrer equation, which was close to the values obtained by PSA, and the same can be analyzed through SEM micrographs. The peak sharpness and intensity authenticate the crystalline nature of as prepared NPs. These findings are supported the fact that hematite nano-crystals may develop preferentially rather than arbitrarily [38, 39].

ATR-FTIR was used to characterize the different stretching modes, vibrational frequency, and functional groups present in the IONPs. Fig 3b demonstrates the infrared spectrum of IONPs in the range of wavelength number 500–4000 cm^-1^ which identifies both functional groups and chemical groups. An ATR-FTIR spectrum of IONPs shows distinct peaks at 3363.74, 2342.13, 2008.88, 1652.92, 1084.87, and 683.44 cm^-1^. The intensity of the absorption band at 3363.74 cm^-1^ is attributed towards H bonds of O-H because of the binding vibration band of chemisorbed on the surface. The strong signals related to the C = C stretch of alkynes are marked between 2000–2500 cm^-1^. Broad peak at 1652.92 cm^-1^ is assigned to the C = O stretch on the IONPs surface. The peak at 1084.87 cm^-1^ indicates the presence of aliphatic amine [40]. Absorption bands found in the region below 1000 cm^-1^ are due to inter-atomic vibration and metal oxides. Moreover, the peak in the region below 700 cm^-1^ is attributed to FeO stretch hence, peak at the absorption band near 684.44 cm^-1^ is due to the Fe-O stretches of Fe_2_O_3_, which indicates the synthesis of iron oxide NPs. These bands of Fe_2_O_3_ molecules vibration were also reported in previous studies [41, 42]. FTIR spectrum of IONPs reveals that natural compounds in the form of functional group attach with NPs due to electrostatic force present in Fe^+2^ metal ions. These interfaces suggest NPs as an ideal candidate to boost plant regeneration and development [43].

The optical properties of NPs are of immense importance in evaluating biological applications. It is imperative to know accurate information about optical features of nanoparticles as these characteristics evaluate their light absorbing potential at room temperature. Fig 3c depicts the UV-visible absorption spectra of the Fe_2_O_3_ solution. The colloidal suspension was visually observed from light reddish-brown to dark reddish-brown and absorption spectra were recorded in the range between 200–800 nm. The absorption peak at 300 nm is characteristic of the α-Fe_2_O_3_-NPs owing to the electronic transition between O and Fe. Bandgap energies of IONPs were calibrated by UV data and utilizing Tauc relation for direct bandgap material (αhν)^2^ = A(hν—E_g_).

Where hν stands for photon energy, E_g_ represents bandgap energy, A is constant and α is the absorption coefficient. The linear examination of the (αhν)^2^ curves up to the energy axis showing bandgap energy of IONPs. The E_g_ value of green synthesized IONPs is found to be 2.77 eV which is apparent from the earlier study [44].

The UV absorption phenomenon is due to surface plasmon resonance which may be result in a red shift or blue shift depending on the morphology of NPs, size, surface charge value, chemistry of solvent, and concentration in the solvent [45]. Surface activation of IONPs is linked to the polarization of metal complex that transferred the resonance in the optical region. The coarse surface of synthesized IONPs is due to the presence of plant natural compounds that are acting as surface capping agents [46]. However, it has been noted that irregular morphology of NPs can enhance their surface area which increases reactivity and boosts their activity in plant regeneration.

TGA analysis demonstrates the thermal behavior as well as capping action of the natural compounds from *C*. *jwarancusa* leaves extract on IONPs (Fig 3d). The change in mass of the IONPs with increase in temperature was examined. Up to 200°C, the weight of IONPs was observed to decrease as a result of evaporation of moisture adsorbed on the surface. The loss observed around 550°C is expectedly due to mass combustion of biomolecules (mainly phenolics and flavonoids) which are acting as surface stabilizing agents around IONPs. The combustion of natural compounds illustrates the successful capping of synthesized IONPs. The weight loss observed around 600°C, due to transition phase of synthesized NPs. After 600°C, the TGA curve becomes parallel to temperature axis, which indicates high stability of IONPs [47].

### Effect of α-Fe_2_O_3_-NPs on callus induction frequency

**Fig 4:**
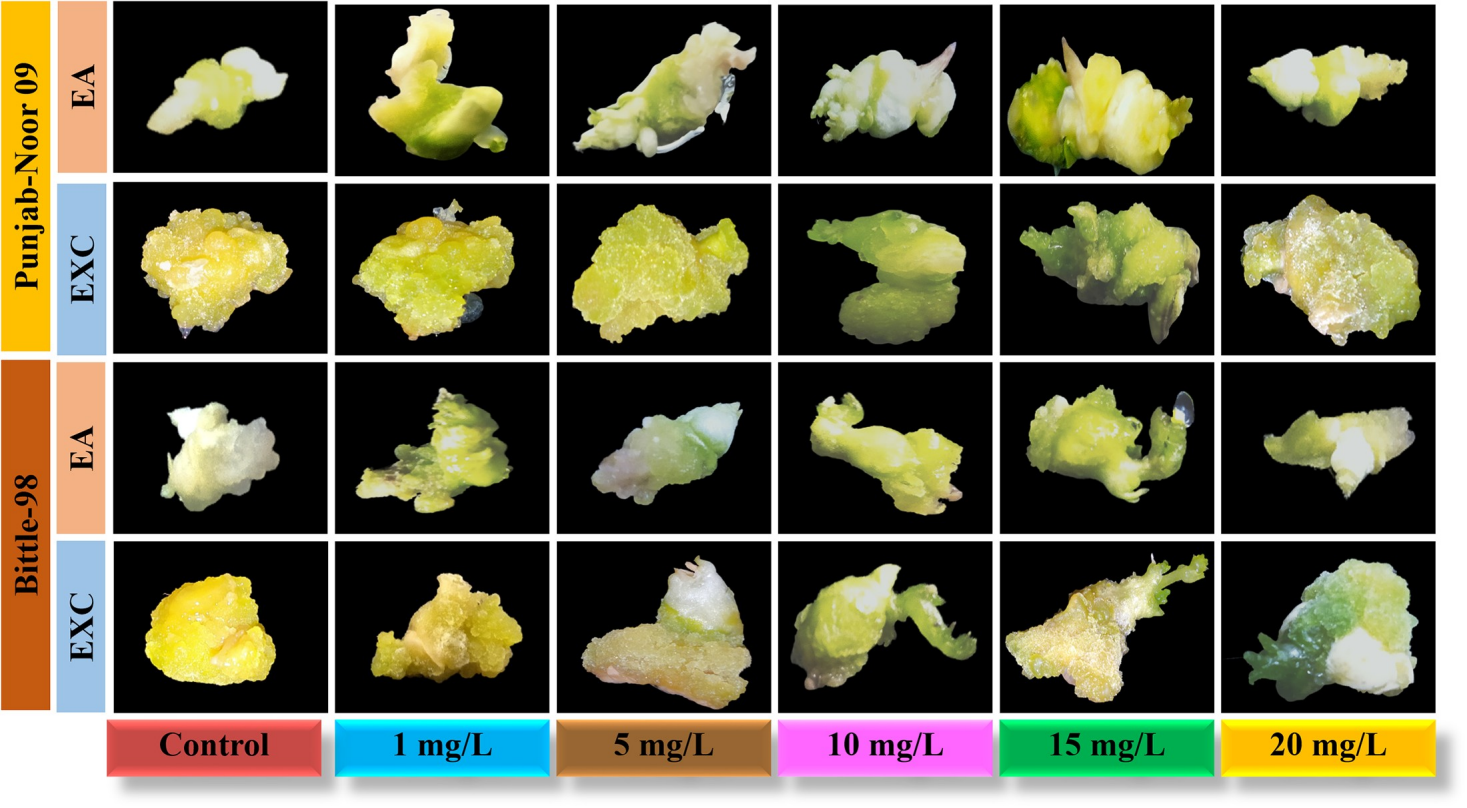
Effect of IONPs (1, 5, 10, 15, 20 mg/L) on callogenesis of chickpea varieties; EA, embryo axes with removed root apex, EXC embryo axes plus attached part of the cotyledon.

In the present study, effect of biosynthesized IONPs from *C*. *jwarancusa* leaves extract was investigated for its efficacy in tissue culture of chickpea. This is the first comprehensive study of IONPs on chickpea tissue culture. Fe play a significant role in different physiological processes including redox reaction, respiration, and chlorophyll biosynthesis [48]. Iron is the most important nutrient for plant metabolism and growth [49] and its deficiency is a common nutritional disorder in different crops, resulting in low yield and productivity. To examine callus induction frequency of chickpea, both varieties Punjab-Noor 09 and Bittle-98 explants, embryo axes (EA) and embryo axes plus cotyledon (EXC) were investigated on MS media without 2,4-D and IONPs. It was examined that both varieties were not capable to induce callus on simple MS medium. Subsequently, the roles of different concentrations of IONPs (1, 5, 10, 15, 20 mg/L) on callus induction of both chickpea varieties were investigated and data is documented in Table 1. Punjab Noor-09 cultivar of chickpea has shown highest callogenesis frequency among both varieties and its EXC explants showed intensively stimulated callus size at 15 mg/L with 96% callus induction frequency followed by 89% at 10 mg/L and 77% at 5 mg/L compared with control 16%. Nevertheless, EA explants of Punjab Noor-09 cultivar showed callus induction frequency 85% at 15 mg/L and 76% at 10 mg/L (Fig 4). Among both tested explants (EXC and EA) of Bittle-98 cultivar of chickpea, the highest callus induction was observed by EXC explants at 15 mg/L of IONPs with 90% increase in callus frequency. Moreover, EA (embryo axes) explants of Bittle-98, showed 78% callogenesis at 15 mg/L and 69% at 10 mg/L concentration while control callus exhibit 34% growth frequency. These findings suggested that IONPs are non-toxic to plants at optimum concentrations (10–15 mg/L) and increase the plant callus induction frequency. The increase in callus induction demonstrate that iron as micronutrient can contribute to plant growth and development [27]. It has been observed [50] that cellular metabolism as a regular function produces hydroxyl radicals, hydrogen peroxide, and superoxide anions. Cell keeps an exquisite balance of reactive oxygen species (ROS) removal and production to prevent cellular oxidative stress, While iron (Fe^+3^, Fe^+2^) have different oxidation states act as ROS scavenger [25], preventing ROS induce mitochondrial destruction and DNA damage inside the cell and eventually increase chickpea growth dynamics. Nanoparticles have shown significant effect on plant growth and regeneration capacity [15]. Nevertheless, we observed a reduction in callus induction frequency in both explants EA (31%) and EXC (44%) in Bittle-98 cultivar, when the concentration of IONPs were increased up to 20 mg/L level. A similar callus inhibition dynamics were observed at 20 mg/L concentration in EA (35%) and EXC (47%) explants of Punjab-Noor 09 cultivar. We conclude that the effect of IONPs was concentration-dependent and an increase in callogenesis was observed between 10–15 mg/L. Further increase in the concentration resulted in the negative response of IONPs which may be due to cell wall injury [51]. It is well reported that higher concentrations of NPs show toxicity in both animals and plant tissues [52]. The IONPs at high concentration can restrict the electron transport chain of chloroplast and mitochondria, which may lead to oxidative burst with high ROS concentration, causing cell death [53], which in turn reduces the callus induction frequency.

**Table 1:** Callus induction frequency of the chickpea cultivars on MS medium at different concentrations of IONPs.

	Bittle-98 variety		Punjab Noor-2009 variety	
Treatments	EA	EXC	EA	EXC
**Control (MS+2,4D)**	20.5±1.1^f^ (34%)	15.2±0.3^g^ (25%)	23.6±0.2^e^ (39%)	9.8±1.5^g^ (16%)
**IONPs (1 mg/L)**	25.2±1.3^e^ (42%)	36.7±1.1^d^ (61%)	30.8±0.7^d^ (51%)	42.2±0.1^c^ (70%)
**IONPs (5 mg/L)**	34.0± 0.9^d^ (56%)	42.1±0.6^c^ (70%)	38.2±2.1^c^ (63%)	46.2±0.8^b^ (77%)
**IONPs (10 mg/L)**	41.5±1.5^c^ (69%)	49.4±1.2^b^ (82%)	45.8±1.2^b^ (76%)	53.5±2.1^ab^ (89%)
**1ONPs (15 mg/L)**	46.9±0.7^b^ (78%)	54.1±2.3^a^ (90%)	51.0±1.9^ab^ (85%)	57.6±1.7^a^ (96%)
**IONPs (20 mg/L)**	18.6±0.2^f^ (31%)	26.4±1.6^e^ (44%)	21.0±1.3^f^ (35%)	28.4±0.9^d^ (47%)

Values show the mean ± standard error (SE) of 20 replicates of each treatment. Values followed by small alphabets stands for the individual values as an average of three replicates (*P* < 0.05). Variable groups (explants, varieties and treatments) that are not represented by same letter are significantly different at (*P* < 0.05). EA, embryo axes with removed root apex, EXC embryo axes plus attached part of cotyledon.

Both variety of explants have shown different response in terms of optimum callus induction frequency at various concentrations of NPs. In Punjab-Noor 09 cultivar, both explants show more callogenesis frequency as compare to Bittle-98 cultivar. Hence, it can be suggested that nanomaterial’s having extremely small size enter in the explants and subsequently may affect genetic reprogramming [54]. Likewise, effect of NPs in plants also depends on particles concentration, plant species, and exposure time [55].

In order to determine the effect of IONPs on organogenesis, calli were shifted to regeneration media supplemented with various concentrations of IONPs ranging from 1, 5, 10, 15 to 20 mg/ L. Following transfer to regenerated media, calli show a stable increase in size on IONPs below 15 mg/L concentration but display no organogenesis or increase in growth on other tested concentrations. Since callus started to increase the size for a longer period (8 weeks) and NPs uptake increased biomass of callus cells, thus, these results encourage us to further study the direct shoot regeneration in chickpeas.

### Effect of α-Fe_2_O_3_-NPs on organogenesis

**Fig 5:**
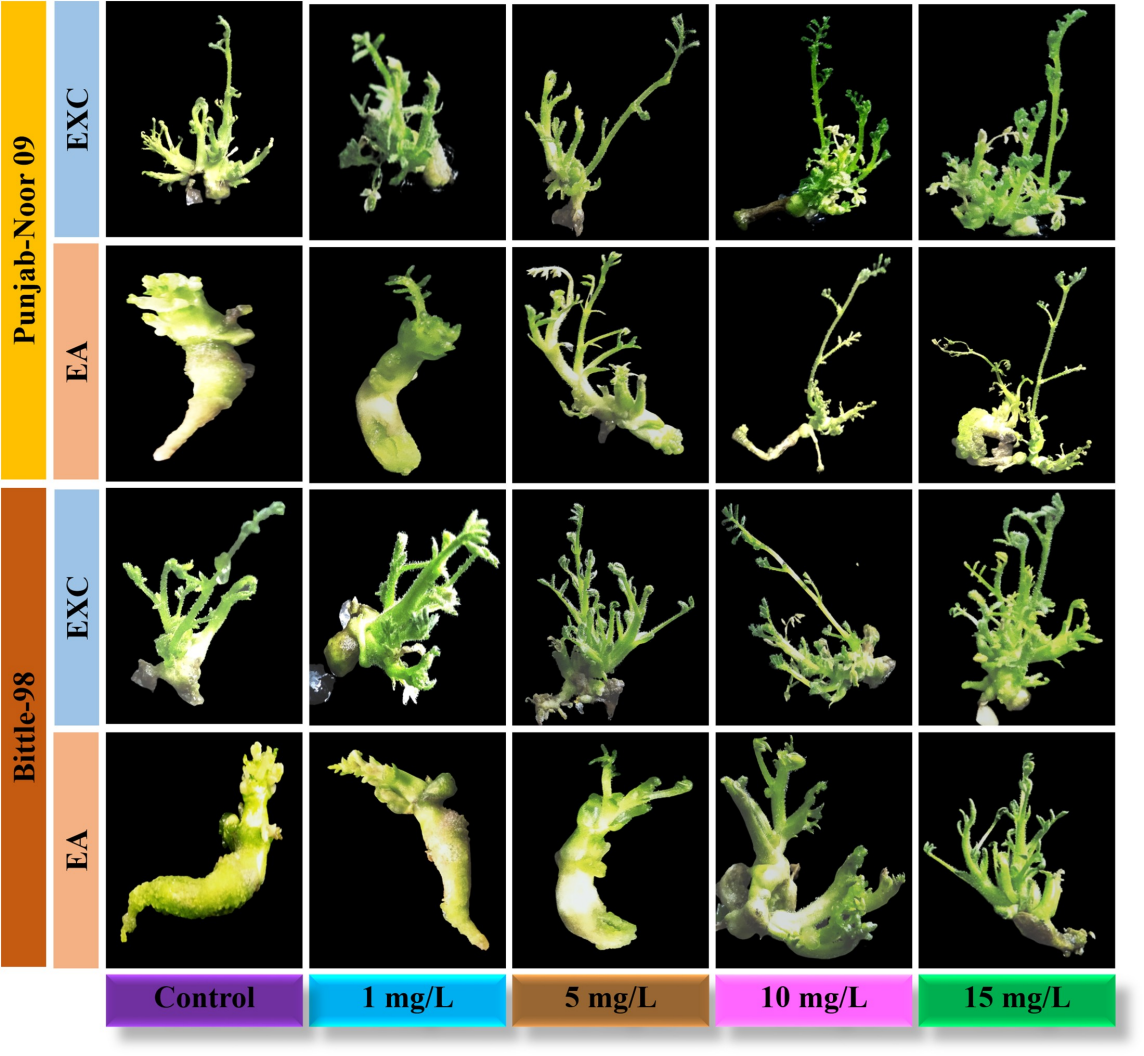
Effect of IONPs (1, 5, 10, and 15 mg/L) on organogenesis of chickpea varieties; EA, embryo axes EXC. Embryo axes plus attached part of cotyledon.

**Fig 6:**
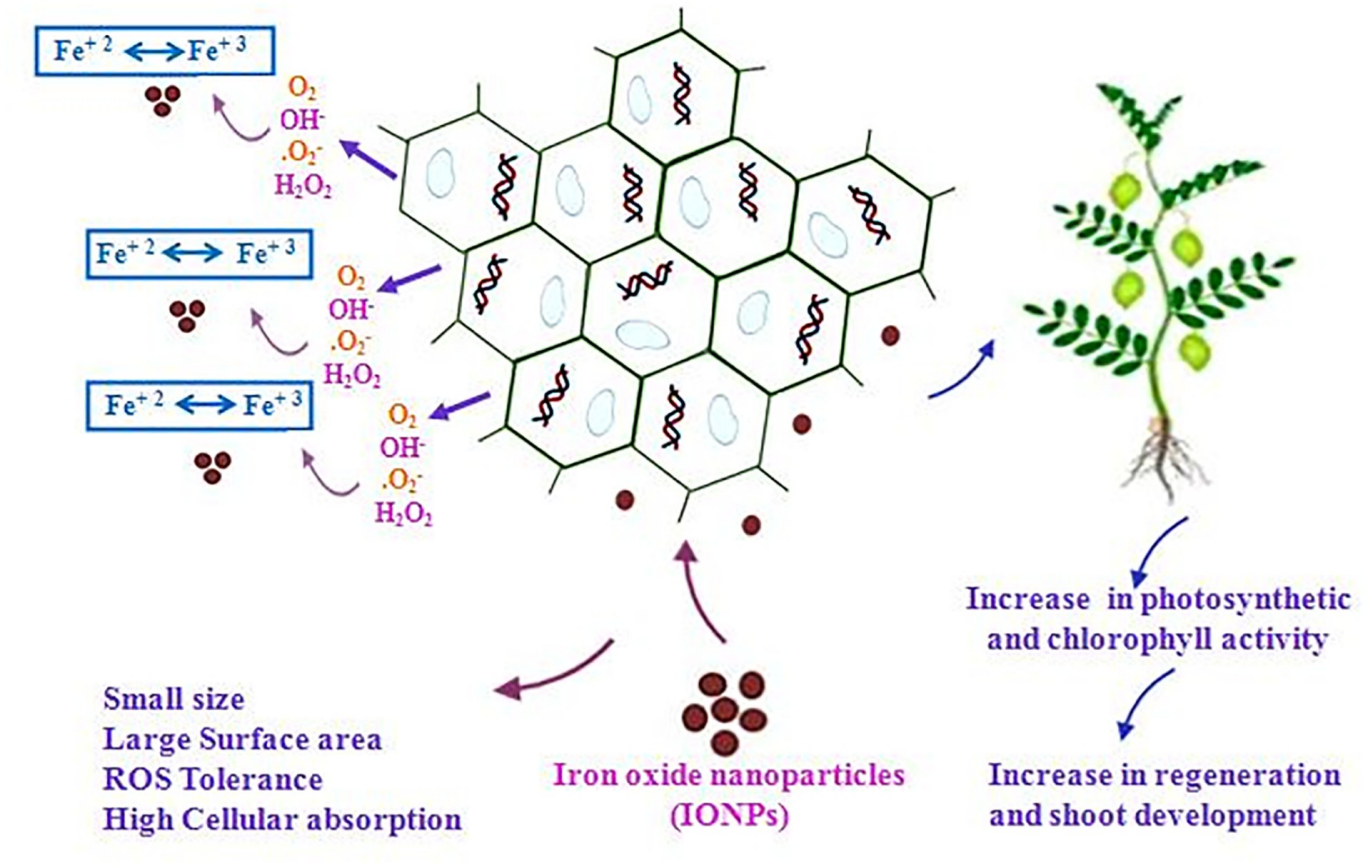
The hypothesized mechanism of action of hematite IONPs as nano-fertilizer to enhance regeneration capability of recalcitrant crops.

For direct shoot regeneration of chickpea, EXC and EA explants of both varieties were examined on MS medium without IONPs and growth hormones. We observed that both explants were unable to show any regeneration, like callus induction. Conversely, shoot regeneration capacity of explants on best-selected regeneration media (SRM-I) was enhanced with the addition of different concentrations of IONPs (Fig 5). Like callogenesis, direct shoot regeneration of EXC explants showed highest frequency of regeneration 88% at 15 mg/L, compared with remaining tested concentration 10 mg/L (84%), 5 mg/L (78%) and 1 mg/L (59%) in Punjab-noor 2009 variety. These results indicate that by increasing the concentration of IONPs, shoot regeneration efficacy is also increased however, at high concentration (20 mg/L) shoot regeneration frequency was decreased to 48%. Similar results are reported by Shankramma et al. [28] in tomato plants which showed reduction in regeneration frequency at high concentration of iron oxide nanoparticles. When EXC explants of Bittle-98 variety were tested against the different concentration of IONPs, they demonstrated similar dose dependent response, 76% shoot regeneration efficiency at 15 mg/L, 69% at 10 mg/L, 60% at 5 mg/L and 46 at 1 mg/L. High frequency of direct shoot regeneration was observed in both variety explants, but best results were achieved at 15 mg/L concentration of IONPs. Iron play a significant role in metabolism such as plant respiration, photosynthesis, electron transfer in a redox reaction, and biosynthesis of chlorophyll and phytohormones regulation [56]. It can therefore be assume that IONPs interact with plant metabolism and they may also adhere to the roots of plants and cause morphological and physiological changes [57]. The possible mechanism of action of NPs on plant cell is that NPs are extremely reactive, therefore, can easily be attached to the plant cell surface and Fe^+3^ released on the cell surface is easily absorbed into cells due to smaller particle size resulting in improved plant growth and development [24]. The overall mechanism of IONPs as nano-fertilizer is proposed in Fig 6. Like callus induction, both explants in Punjab noor-2009 variety showed more regeneration frequency as compare to Bittle-98. The difference in shoot regeneration capability was observed among them (Table 2), however, compared with control, both varieties showed exceptional regeneration response. This shows that nanoparticle activity is directly related to the growth prospective of genotypes [58].

**Table 2:** Effect of different concentrations of IONPs on direct shoot regeneration frequency of chickpea varieties.

	Bittle-98 variety		Punjab Noor-2009 variety	
Treatments	EA	EXC	EA	EXC
**Control (MS+BAP)**	12.6±1.5^g^ (21%)	19.2±2.4^f^ (32%)	21.1±1.3^e^ (35%)	23.5±0.5^e^ (39%)
**IONPs (1 mg/L)**	32.1± 0.7^d^ (53%)	27.6±0.9^e^ (46%)	33.0±0.5^d^ (55%)	35.5±0.9^d^ (59%)
**IONPs (5 mg/L)**	39.2±1.1^c^ (65%)	36.0±1.5^d^ (60%)	42.1±1.0^b^ (70%)	46.8±2.6^ab^ (78%)
**IONPs (10 m g/L)**	42.6±0.0^b^ (71%)	41.5±1.8^b^ (69%)	45.0±2.2^ab^ (75%)	50.5±0.1^a^ (84%)
**1ONPs (15 mg/L)**	47.4.0±0.4^a^ (79%)	45.7±2.5^ab^ (76%)	51.6±0.9^a^ (86%)	53.0±1.5^a^ (88%)
**IONPs (20 mg/L)**	24.1±2.1^e^ (40%)	00.0±0.0 (0%)	27.1±1.8^e^ (45%)	29.0±1.8^d^ (48%)

Data followed by small alphabets stand for the individual as an average of three replicates (*P* < 0.05). Each replicate consists of 20 treatments. EA, embryo axes with removed root apex, EXC embryo axes plus attached part of the cotyledon.

### Effect of α-Fe_2_O_3_-NPs on shoot elongation and root induction

**Fig 7:**
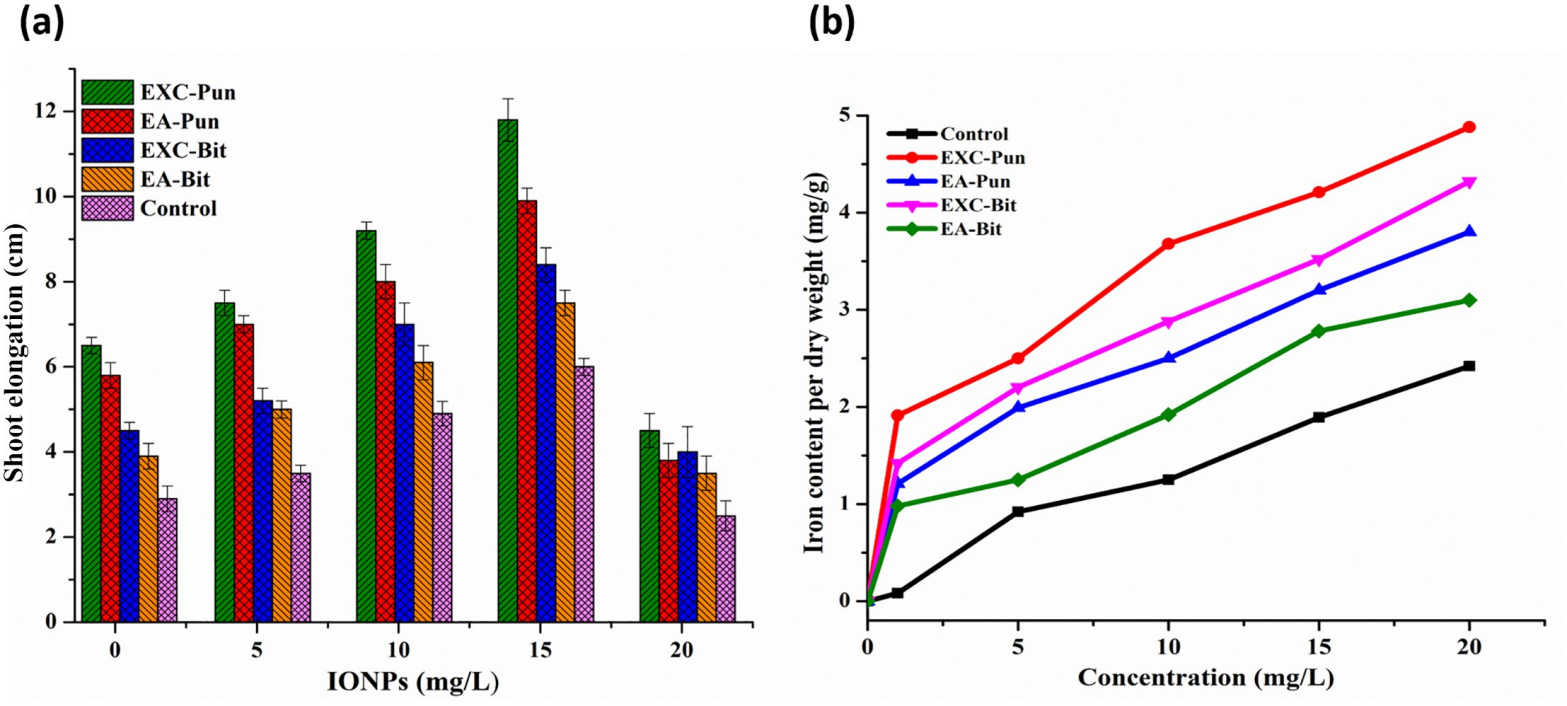
(a) Effect of different concentrations of IONPs on shoot length of proliferating shoots from different explants; EXC and EA, and (b) Iron content per dry weight of regenerated chickpea plantlets.

About 2.2 cm shoot pieces were separated carefully from both variety explants and shifted to shoot elongation (SE) media with different concentrations of IONPs to observe shoot elongation (Table 3). The shoot length of each explant was recorded after 14 days of incubation. Our results indicated that EXC explants of Punjab-noor 09 variety induce highest shoot elongation followed by EA explants of the same variety. Mainly, the healthy and branched shoots produce on 15 mg/L concentration of IONPs showed a maximum length of 11.8±0.5 cm and 9.9±0.3 cm respectively (Fig 7a).

**Table 3:** Effect of different concentrations of IONPs on root induction frequency of chickpea varieties.

	Bittle-98 variety		Punjab Noor-2009 variety	
Treatments	EA	EXC	EA	EXC
**Control (MS+IBA)**	11.0±0.8^a^ (18%)	13.2±0.5^ab^ (22%)	10.2±0.0^a^ (16%)	19.3±0.2^ab^ (32%)
**IONPs (1 mg/L)**	21.6± 0.6^ab^ (36%)	29.5±2.1^b^ (49%)	24.7±1.7^b^ (41%)	33.1±1.2^c^ (55%)
**IONPs (5 mg/L)**	28.2±2.1^b^ (47%)	36.0±1.6^c^ (60%)	31.4±1.5^bc^ (52%)	37.2±1.0^c^ (62%)
**IONPs (10 mg/L)**	35.0±0.7^c^ (58%)	40.2±1.8^cd^ (67)	38.5±0.8^cd^ (64%)	42.6±1.3^d^ (71%)
**1ONPs (15 mg/L)**	41.5±1.5^cd^ (69%)	47.0±2.6^e^ (78%)	45.0±1.2^d^ (75%)	49.8±0.9^e^ (83%)

Values show the mean ± standard error (SE) of 20 replicates each treatment. Values followed by small alphabets stand for the individual values as an average of three replicates (*P* < 0.05). Variable groups (explants, varieties and treatments) that are not represented by the same letter are significantly different (*P* < 0.05). EA, embryo axes with removed root apex, EXC embryo axes plus attached part of cotyledon.

Rooting is considered as one of the key obstacles for efficient regeneration of chickpea and scientists have developed different strategies to overcome this difficulty. Many of them utilize micro-grafting techniques, some researchers manipulated the rooting media (decrease salt concentrations), and others used pulse treatment with liquid media having auxins [59]. Although these methods increase root efficiency in chickpea, nonetheless, they are difficult to handle and are time consuming. A reliable chickpea regeneration system has not been available yet, because of the fact that it owns recalcitrant nature to regeneration [60]. Iron is used as an enzyme cofactor and being an indispensable element for the photosynthetic process. In recent years, iron based nanomaterial are used as fertilizers to enhance crop yield [61]. However, to the best of our knowledge, no attention has been paid earlier to examine the effect of IONPs on the type of explants utilizing to initiate the regeneration process on root induction. We tried our level best to study this aspect by taking regenerated shoots from each type of explants (EA and EXC) and assessed their rooting frequency. Results revealed that embryo axes with adjacent cotyledon explants (EXC) show higher rooting pattern than shoot regenerated from other type embryo axes (EA) explants. Similar finding is also reported by Amer et al. [59] suggesting that type of explants has a direct effect on shoots and root regeneration ability. After 10 days of incubation of regenerated shoots with different concentrations of IONPs (1–15 mg/L), highest root induction was observed in EXC explants of Punjab-Noor variety; 83% root induction at 15 mg/L concentration, 71% at 10 mg/L, 62% at 5 mg/L, while 32% root induction was observed in control plants (Table 3). EA explants of Punjab Noor-09 variety show 75% root induction at 15 mg/L. While Bittle 98 variety, both explants show 78% (EXC) and 69% (EA) root induction at 15 mg/L concentration. The maximum rooting response with healthy roots and root hairs were observed at a concentration of 15 mg/L followed by 10 mg/L. Phenotypic analysis of roots revealed that IONPs (1, 5, 10, 15 mg/L) treated explants displayed a concentration-dependent response in root length and branching. Our results proved that different concentrations of IONPs are highly effective for root induction in chickpea plants and this study is a contribution towards improving regeneration dynamics and transformation system of chickpea particularly and legumes in general.

### Iron content in the regenerated chickpeas

Iron content in regenerated chickpea plantlets were quantified by inductively coupled plasma-optical emission spectrometry. IONPs treated chickpea explants showed significantly higher iron content in regenerated plantlets (Fig 7b). Results demonstrate that in all experimental group, IONPs were translocated in regenerated shoots. However, Punjab noor-09 EXC explant shoots at 15 mg/L concentration of IONPs possess significant amount of iron 4.21 mg/g, whiles control regenerated shoots contained 1.89 mg/g iron content. These findings are in line with previous results obtained from callogenesis (Fig 4) and shoot regeneration (Fig 5). It seems that iron oxide nanoparticles with 50 nm size, contributed well to enhancing regeneration and growth in chickpea explants. Iron is an important micronutrient involves in a different physiological reaction and is an imperative component of chlorophyll [62]. Our results also reveal that iron concentration has been increased in chickpea shoots with increasing concentrations of NPs. This could be due to the biomineralization and internalization of localized iron in different parts of the chickpea plant. The results suggest that smaller particle size can dissolve iron more effectively in plantlets and more iron is provided to chickpea regenerated plants, resulting in rapid plant growth and development. Thus, iron can be used as a nano-fertilizer to enhance plant growth, yield and productivity.

## Conclusion

The study concludes that green synthesis of IONPs has proved to be a more biocompatible, efficient, sustainable, and economical method, which can be used efficaciously in plant tissue culture studies. Phytochemical enrich *C*. *jwarancusa* leaves extract have effectively tailored NPs through strong capping and stabilizing agents. SEM revealed green synthesized IONPs have rod-shaped morphology with a size of 50±0.2 nm while XRD confirms the highly crystalline nature of α-Fe_2_O_3_. ATR-FTIR confirms pure chemical behavior of green fabricated NPs. IONPs at optimum dose increase callogenesis, regeneration dynamics, shoot elongation and root induction in chickpea plantlets. The regeneration potential of chickpea gives promising results and keep plant seedlings infection-free by provide an excellent environment for rapid regeneration and growth. Overall, this study shows an increase in growth parameters of chickpea plant using green synthesized IONPs. These observations encourage that green fabricated IONPs could be used in agronomic crops for environmental sustainability. Nevertheless, further studies at molecular level are needed for further considerations and the use of IONPs for more crop productivity, abiotic and biotic stress resistance.

## Supporting information

S1 File (DOCX)
