# Supplementary material for: Biogenic iron oxide nanoparticles enhance callogenesis and regeneration pattern of recalcitrant Cicer arietinum L
Source: PLoS One. 2020 Dec 1;15(12):e0242829. doi: 10.1371/journal.pone.0242829 (PMC7707474; doi:10.1371/journal.pone.0242829)
Supplement: S1 File — (DOCX) [file pone.0242829.s001.docx]

**Biosynthesized nano-formulated Iron Oxide media enhance callogenesis and regeneration pattern of recalcitrant *Cicer arietinum* L.**

Samra Irum^1^, Nyla Jabeen^1*^, Khawaja Shafique Ahmad^2*^, Saima Shafique^1^, Talha Farooq Khan^3^, Hina Gul^4^, Sadaf Anwaar^1^, Nuzhat Imam Shah^5^, Ansar Mehmood^2^, Syed Zaheer Hussain^6^

^1^Applied Biotechnology and Genetic Engineering Lab, Department of Biological Sciences, International Islamic University, Islamabad 44000, Pakistan

^2^Department of Botany, University of Poonch, Rawalakot (UPR), 12350, Azad Jammu and Kashmir, Pakistan

^3^Department of Materials Science & Engineering, Institute of Space Technology Islamabad 44000, Pakistan

^4^University Institute of Biochemistry and Biotechnology, PMAS Arid Agriculture University, Rawalpindi, Pakistan

^5^Department of Microbiology, Hazara University, Mansehra, Pakistan

^6^Department of Biological Sciences, Quaid-i-Azam University, Islamabad, Pakistan

**Correspondence**

^1*^Nyla Jabeen

nylajabeen8@gmail.com

^2*^Khawaja Shafique Ahmad

[shafiquebot@yahoo.com](mailto:shafiquebot@yahoo.com)

[ahmadks@upr.edu.pk](mailto:ahmadks@upr.edu.pk)

ORCID: <0000-0001-8814-9878>

**Abstract**

This study is the first report on the biosynthesized hematite iron oxide nanoparticles (IONPs) which mediate in-vitro callus induction and shoot regeneration in economically important recalcitrant chickpea crop (*Cicer arietinum* L.). Here, we used leaf extract of *Cymbopogon jwarancusa* in the green synthesis of IONPs in order to achieve a better biocompatibility. The secondary metabolites present in the leaf extract served as both reducing and capping agents in fabrication process of IONPs. Field emission scanning electron microscope (FE-SEM) analysis revealed rods like surface morphology of IONPs with an average diameter of 50±0.2 nm. Energy-dispersive X-ray spectroscopy (EDS) depicted the major peak of iron (69.84%) and a minor peak of oxygen (30.16%). We used X-ray diffractometry (XRD) and attenuated total reflectance-Fourier transform infrared (ATR-FTIR) to validate the phase purity of synthesized α-hematite IONPs. UV-visible absorption spectrophotometry showed the formation of single-phase stable structures. Thermo-gravimetric analysis (TGA) displayed thermal loss of organic capping around nanoparticles at 400-500°C and confirmed their stabilization. The biosynthesized IONPs revealed very promising results in callus induction, shoot regeneration and root induction of chickpea plants. Both chickpea varieties Punjab-Noor 09 and Bittle-98 explants, Embryo axes (EA) and Embryo axes plus adjacent part of cotyledon (EXC) demonstrated dose-dependent response. Among all explants, EXC of Punjab-Noor variety showed the highest callogenesis (96%) and shoot regeneration frequency (88%), while root induction frequency was also increased to 83% in EXC explants. Additionally, the results revealed a significant enhancement in regenerated plants (4.88 mg dry g^-1^) in comparison with the control treatment (2.42 mg dry g^-1^). We conclude that IONPs improve crop nutrition at nanoscale and keep chickpea plantlets infection free by providing an optimum environment for rapid growth and development. Thus, IONPs synthesized through green process can be utilized in tissue culture studies in other important recalcitrant crops.

**Keywords:** Chickpea; *Cicer arietinum* L; Iron oxide nanoparticles; Callogenesis; Regeneration; Tissue culture

**Introduction**

Chickpea (*Cicer arietinum* L.) is one of the most important legume crops in the world with an annual yield of 11.6 million tons [1]. It plays a major role in the diet of millions of people in developing countries and is therefore sometimes referred to as the meat of the poor. [2]. Pakistan is the fourth-largest producer of chickpeas in South Asia [3]. Since chickpeas are rich source of proteins, carbohydrates, vitamins and minerals, they provide diversified human health benefits and nutritional values [4]. The crop is used in agriculture system for sustainable production because it directly provides biologically fixed nitrogen, and maintains soil productivity [5]. Different chickpea derivatives such as culled chickpea, low-grade pea, chickpea pod husks, and hay are widely used as protein-rich feed in animal diet and chickpea straw as alternative forage for ruminants [6]. The demand for high quality chickpeas is increasing steadily with increasing population need of the Indian subcontinents and is expected to reach 17.0 Mt in 2020 [7]. The global chickpea yield does not meet the present demand due to various biotic and abiotic factors [8]. Fungal diseases mainly Ascochyta blight and Fusarium wilt are the major biotic constraints involved in significant yield loss of chickpea crop worldwide [9].

Environmental stresses cause serious damage to the agricultural crops thereby hindering the crop productivity. To overcome these production restraints, it imperative to use advance biotechnological approaches for the improvement of legumes and understand the genetics of multifaceted traits [10]. Plant tissue culture technique is used to screen plantlets and provide opportunity to study the different aspects of plant growth and development. Additionally, this technique has become vital for the selection of plant tolerance to several abiotic and biotic stresses [11]. The biggest advantage of in-vitro micropropagation of plants is the production of more number of plants in a very short time under controlled environmental conditions [12].

Nanotechnology is an emerging field that has novel applications in agriculture and plant biotechnology [13]. Nanoparticles (NPs) can be synthesized from different organic and inorganic materials. In recent times, green synthesis of metallic NPs have received much attention due to their wide applications in various fields of science and technology including electronics to structural engineering and agriculture to medicine. [14]. Typically, nanoparticles have small size (less than 100 nm), high surface area, and eco-friendly nature [15]. In the last decade, nanomaterials have occupied a prominent place in agriculture sector because of their useful applications in agribusiness [16]. Metallic nanomaterials enhance the productivity of crops, accelerate plant germination and regeneration capacity, and develop crop resistance against various biotic and abiotic stresses. It is, therefore, imperative to use micronutrients the form of nanoparticles to increase the crop productivity [17].

Iron oxide nanoparticles (IONPs) are one of the most important oxides in the field of nanomaterials. Previous studies have shown that IONPs have potential application as crop fertilizers due to their physio-chemical properties [18,19]. Iron oxide NPs exist in a diversified polymorphic form including maghemite (γ-Fe_2_O_3_), goethite FeO(OH), and hematite (α-Fe_2_O_3_) [20]. Among all of them, hematite (α-Fe_2_O_3_) is thermodynamically and chemically more stable form of iron-oxide nanoparticles because of its rhombohedral geometry and centered hexagonal structure with dense-packed O_2_ lattice [21]. Hematite nanoparticles can be used as starting material in the synthesis of maghemite and magnetite nanoparticles. Furthermore, Fe_2_O_3_-NPs have wide applications in agriculture, cosmetics, biomedicine, diagnostics, material engineering, and bioremediation [22,23]. Iron oxide nanoparticles synthesized from green route are nontoxic to humans and are more biocompatible when compared to their chemically synthesized counterparts [24]. In addition to improve in-vitro development of strawberry plantlets against water stress, IONPs enhance growth in the corn, tomato, rapeseed, and sorghum [25,26,27].

Iron is an important micronutrient, involves in chlorophyll formation and other cellular reactions in plants. Fe plays significant role in plant growth and metabolism, phytohormonal regulation mainly auxins, carbohydrate metabolism, protein synthesis, and stress-related response [28]. In Plants, iron uptake is highly regulated through apoplastic pathway via roots in order to supply amounts sufficient for optimal growth [29].

Chickpea is one of the most important pulse crops with a diverse array of potential nutritional and health benefits. As a model plant, chickpea has been increasingly investigated as a mean to improve agronomic traits. Nevertheless, to date, no research has been made on in-vitro tissue culture of recalcitrant chickpea crop using nanoparticles. Keeping in view the existing gap in our knowledge, the current study focuses on the synthesis and characterization of iron oxide nanoparticles utilizing *C. jwarancusa* aqueous leaves extract. Further, biosynthesized Fe-enrich hematite (α-Fe_2_O_3_) nanoparticles are evaluated to boost callogenesis and shoot regeneration dynamics in recalcitrant chickpea cultivars. It is believed that these findings will help researchers to mitigate the scarcity of food supply and related malnutrition by improving agricultural productivity of economically important legume crops.

**Materials and methods**

**Green synthesis of Iron oxide nanoparticles (IONPs)**

*Cymbopogon jwarancusa* leaf extract was used for the synthesis of IONPs ascribing its pharmacological potential. Briefly, fresh plant sample of *C. jwarancusa* grass were collected from the northern region of the Nara desert of Pakistan and authenticated by an expert taxonomist at the department. Firstly, fresh leaves were washed three times with double distilled water (d.dH_2_O) to remove dust particles and other contaminated organic materials. To avoid dissociation of bioactive compounds, collected plant leaves were first shade dried at room temperature later, dried leaves were crushed into a fine powder using Wiley Mill grinder (Thomas Scientific ED-5). For extraction purpose, 30 g of fine leaf powder was dissolved into 300 ml of d.dH_2_O (Mili-Q Merck^TM^) in an Erlenmeyer flask and kept on boiling for 30 min at 100ºC. In the next step, flask was placed on orbital shaking incubator set to 37ºC at 100 rpm for overnight to obtain phytochemical enrich extract. Subsequently, leaves extract was vacuum filtered through Whatman^TM^ 41 filter paper (GE Health care, 20 µm) and filtrate extract was stored at 4ºC in the refrigerator for further use [30].

For green synthesis of IONPs, Iron (III) nitrate nonhydrate (Fe(NO_3_)_3_^.^9H_2_O), (Sigma Aldrich) was used as a precursor of iron salt (Fe^+2^). Briefly, 4.86 g salt was added to 300 ml of *C. jwarancusa* leave extract (pH 5.7). The reaction solution was stirred on hot plate magnetic stirrer (IKA^TM^) heated to 70ºC at 1200 rpm for 2 h. Following, reaction mixture was centrifuged at 10,000 rpm (GR-BioTek) for 15 min till brownish-red color pellet of IONPs was collected. The pellet was then washed thrice with d.dH_2_O to remove impurities and secondary metabolites, thereafter dehydrated in hot air drying oven (Memmert) at 65ºC for 6 h (Fig 1). In order to achieve better crystalline structures, IONPs were calcinated inside Gallenkamp Muffle-furnace for 2 h at 500ºC [31].

**Fig 1**. Stepwise schematic protocol for green synthesized IONPs using leaves extract of *C. jwarancusa*.

**Morphological characterization of IONPs**

Field emission scanning electron microscopy (FESEM, TESCAN, MIRA3) was used for surface morphological characterization at 20 kV with a counting rate of 2838 and equipped with energy-dispersive X-ray spectroscopy (EDS, Oxford) for chemical composition analysis of green synthesized IONPs. The FE-SEM micrographs of IONPs were taken at 10KX, 25KX, 50KX and, 100KX resolutions. Particle size analysis was performed using particle size analyzer (PSA, MALVERN master sizer hydro 3000).

**Structural, vibrational, optical, and thermogravimetric analysis of IONPs**

Crystallographic parameters and phase purity of IONPs were characterized using X-ray powder diffraction (XRD) spectroscopy (GNR analytical instruments groups EXPLORER RD 50 1,5 mm Pb 110 KV 1879619). The scanning rate of 2 µ angles/min with an operating voltage of 40 kV and 30 mA current was supplied in an ambient environment. The diffraction range of 2θ was set from 20º to 80º and average crystalline size (D) was evaluated using Debye Scherrer’s [D=0.9λ/βcos] equation.

For vibrational characterization of IONPs and functional groups involve in capping and reduction of green synthesized IONPs, ATR-Fourier transform infrared spectroscopy (ATR-FT-IR, NICOLET iS10, Thermo Scientific) was performed within the spectral range of 500-4000 cm^-1^ and obtained peak values were compared with the standard database.

The optical characterization of biosynthesized IONPs was performed through UV-visible spectroscopy in the range between 200-800 nm (Shimadzu spectrophotometer UV-1800, Kyoto, Japan) under ambient conditions. For this, 1 mg of IONPs was dissolved in deionized water and suspension was sonicated for 25 min. Subsequently, sample was placed in the UV-visible spectrophotometer. Moreover, Thermo Gravimetric Analysis (TGA, 1 STAR^e^ system, METTLER TOLEDO, USA) was carried to analyze the progressive weight loss, thermal stability, and bimolecular capping around green fabricated IONPs. The increasing temperature in TGA was set between 25-1000ºC with an increment of 10ºC per minute.

**Collection of Plant material and explants preparation**

Seeds of two Pakistani chickpea cultivars namely, Punjab-Noor 09 (Kabuli type) and Bittle-98 (Desi variety) were obtained from National Agriculture and Research center (NARC), Islamabad Pakistan. Uniform seeds were surface sterilized with 70% ethanol for 3 min and then washed with distilled water. Next, seeds were sterilized with 40 % of commercial Clorox for 20 min and rinse three times with sterile distilled water (Mili-Q, Merck^Tm^). Sterilized chickpea seeds were soaked in sterile distilled water for 16 h under aseptic conditions. Next day, the soaked chickpea seeds were decoated and two sets of explants were designed. One set of embryo axes (EA) with removed root apex and the second set with embryo axes (EXC) plus adjacent part of the cotyledon with removed root apex was used as explants.

**Media Preparation**

Murashige and Skoog medium (MS) 4.43 g/L, with pH 5.8, vitamins (I00X) along 3 % (w/v) of sucrose as carbon source was thoroughly mixed. About 3% Gellan-gum (PhytoTech Labs) was added to the media as a solidifying agent and autoclaved at 121ºC for 20 min. Different concentrations of IONPs (1, 5, 10, 15, 20 mg L^-1^) were added to autoclaved MS media. To avoid agglutination of nanoparticles at the bottom of the flask, the media was allowed to cool up to 45ºC. In the next step, media flask was kept at room temperature till solidification and all the work was carried under sterilized conditions.

**Influence of IONPs on callus induction**

For callus induction, MS media with 2,4-D (1.5 mg/L of 2,4-Dichlorophenoxyacetic acid) was used and supplemented with various concentrations of IONPs (1, 5, 10, 15, 20 mg L^-1^) and without IONPs as a control. The culture tubes were set aside in dark and white fluorescent light (16/8 h photoperiod) conditions at 25±2ºC for 14 days. After a cultivation period of 13-16 days, embryogenic callus induced from explants were achieved [32]. Callus induction frequency was calculated using the following formula:

$$Callus Induction frequency \left( \% \right)=\frac{No.of Calli produced by explant}{No.of explant inoculated} \times100 (I)$$

**Influence of IONPs on organogenesis**

Both varieties of explants (EA+EXC) were investigated on shoot regeneration media (SRM) containing different concentrations of cytokinin (BAP) and kinetin (Kn). SRMI (MS+ 0.5mg/L BAP), SRMII (MS+ 1 mg/L BAP+ 0.5 mg/L Kn) supplemented with different concentrations of IONPs (1-20 mg L^-1^) and without IONPs as control. The regeneration frequency was observed as the percentage of chickpea explants respond to the development of shoot after 14 days [32]. The regeneration frequency was calculated using the formula given below:

$$Regeneration Frequency \left( \% \right)=\frac{No.of expalnts regenerated into plantlets}{No.of explants inoculatedfor regeneration} \times100 (II)$$

**Influence of IONPs on shoot elongation**

Shoot elongation is a necessary step in organogenesis as it gives strength to the plant stem. Shoots/explants growing on shoot regeneration media were carefully separated from an intact basal part. Healthy shoots were then selected and shifted to shoot elongation media (SE). Different concentrations of IONPs (1-20 mg L^-1^) with a combination of Thiodiazuron (TDZ, 1 mg L^-1^) were used for shoot elongation. The average length of shoot induced on SE was determined after 2 weeks post-transfer to observe the best shoot elongation. The cultures were kept at 25±2ºC under white fluorescent light for 16 h photoperiod.

**Influence of IONPs on root induction**

To assess the effect of IONPs on rooting, healthy shoots were transferred to root induction media (RIM). The regenerated shoots were cultured on MS media supplemented with different concentrations of IONPs (1-20 mg L^-1^) along with indole 3-butyric acid (IBA, 1.5 mg L^1^). Growth conditions were same as 25±2ºC for 16 h photoperiod under white fluorescent light.

**Determination of iron content in regenerated chickpeas**

The regenerated healthy fresh shoots were carefully harvested, washed with distilled water, dehydrated in oven at 60ºC for ^˷^ 18 h, and then homogenized. These homogenized shoots were then digested in the acid mixture having concentrated H_2_SO_4_, HNO_3,_ and HCIO_4_ (60%) acids (1:3:1) for 24 h in Single Reaction Chamber Microwave digestion system (MILESTONE, LabTech, Italy). Later, complete digestion final volume of 10 ml was made with distilled H_2_O. The amount of iron present in sample solution was determined via inductively coupled plasma-optical emission spectrometry (OES-ICAP6500, Thermo Scientific) and is expressed as µg g^-1^ (dry weight) of plant tissue [33].

**Statistical analysis**

All the data were examined statistically using OriginPro^TM^-8.5 software. All the experiments were repeated thrice (n=3) and each replication has 20 observation.

**Results and discussion**

**Morphological and chemical properties of IONPs**

The morphology of green synthesized IONPs were examined through a Field emission scanning electron microscope (FE-SEM) while its chemical composition was confirmed through energy-dispersive X-ray spectroscopy (EDS) as shown in Fig 2. IONPs prepared through green method have highly crystalline needle-like shape and rod-like morphology, henceforth, these NPs will be referred as hematite Fe_2_O_3_ nanorods. Fig 2 (a-d) presents SEM micrographs of IONPs nanorods at an increasing magnification of 5µm, 2 µm, 1 µm, and 500 nm respectively and individual IONPs nanorods can be observed at higher magnification. The diameter of nanorods ranged between 38-79 nm. A high concentration of nanoparticles is ^˷^50 nm in size, which is in agreement with the XRD data. EDS spectrum in Fig 2(e) depicts that Fe_2_O_3_ nanorods are of high purity since only two peaks of iron and oxygen was observed. The major peak indicates the presence of of Fe (69.84 wt%) while minor refers to the remaining oxygen content (30.16 wt%). The current findings are supported by previous reports; where Al-Ruqeishi et al. [34] and Aisida et al. [35] reported similar green synthesized iron oxide nanorods like morphology. While modifying the pH results in a change of surface charge of secondary metabolites which affect the binding potential and reducing capability of metal ions in the synthesis of nanoparticles [36]. In our findings, Fe_2_O_3_ nanorods-like morphology of green synthesized IONPs indicates successful capping of natural compounds from *C. jwarancusa* extracts.

The average size of IONPs was measured using particle size analyzer (PSA). As shown in Fig 2(f), we observed two different peaks, one at an average particle size of about 50nm while the other at around 560 nm. By interconnecting the data obtained through SEM with particle size analysis, we can suggest that the smaller peak gives a measure of the average diameter of the Fe_2_O_3_ nanorods, while the larger peak resulted from the measurement along the length of the Fe_2_O_3_ nanorods. These results perfectly matched with data generated by XRD analysis.

**Fig 2.** Field emission scanning electron microscope (FE-SEM) micrographs of IONPs showing nanorods at a resolution of (a) 10 KX (b) 25 KX (c) 50 KX (d) 100 KX (e) Particle size distribution of IONPs.

**Crystallographic, vibrational, optical and thermal behavior of IONPs**

The crystalline nature and phase purity of IONPs was investigated through X-ray diffraction method. our data depicted that Fe_2_O_3_ phase transformation occurs during calcination between 300 to 400ºC and a sudden increase in the phase (γ → α) occurs when calcination temperature raise above 400ºC. Consequently, pure α-Fe_2_O_3_ powder can be obtained through calcination above 500ºC. Fig 3(a) illustrates the crystallographic nature of IONPs in the range between 20º-80º. The XRD pattern clearly indicates eleven distinct peaks indexed to (012), (104), (110), (006), (113), (202), (024), (116), (018), (214), and (300), corresponding to the 2θ values of 24.1, 33.2, 35.7, 38.9, 40.9, 49.4, 54.1, 57.6, 62.6, and 64.0º respectively (Fig 3a). The comparatively higher (104) peak reveals crystal planes of rhombohedral and suggests ideal orientation of the hematite crystallites. All noted peak intensity scans are indexed to the rhombohedral α-Fe_2_O_3_. The same crystallographic pattern of IONPs was also observed by Jacob et al. [37] and Trpkov et al. [38]. The average size of crystalline α-Fe_2_O_3_ is 50±0.2 nm which is close to the values obtained by PSA, and the same can be analyzed through SEM micrograph. All the peaks in spectra are in accordance with JCPDS card no. 79-1741. The peak sharpness and intensity authenticate the crystalline nature of prepared NPs and the spectrum indicates high phase purity. These findings are supported the fact that hematite nano-crystals may develop preferentially rather than arbitrarily [39,40].

ATR-FTIR was used to characterize the different stretching modes, vibrational frequency, and functional groups present in plant extracts and IONPs responsible for the reduction of Fe^+3^ ions to nanoparticles and stabilize the green synthesized nanoparticles. Fig 3(b) demonstrates the infrared spectrum of IONPs in the range of wavelength number 500-4000 cm^-1^ which identifies both functional groups and chemical groups. An ATR-FTIR spectrum of IONPs shows distinct peaks at 3363.74, 2342.13, 2008.88, 1652.92, 1084.87, and 683.44 cm^-1^. The intensity of the absorption band at 3363.74 cm^-1^ is attributed towards H bonds of O-H because of the binding vibration band of chemisorbed. The strong signals related to the C=C stretch of alkynes are marked between 2000-2500 cm^-1^. Broad peak at 1652.92 cm^-1^ is assigned to the C=O stretch on the IONPs surface. The peak at 1084.87 cm^-1^ indicates the presence of aliphatic amine [41]. Absorption bands found in the region below 1000 cm^-1^ are due to inter-atomic vibration and metal oxides. Moreover, the peak in the region below 700 cm ^-1^ is attributed to FeO stretch hence, peak at the absorption band near 684.44 cm^-1^ is due to the Fe-O stretches of Fe_2_O_3_, which indicates the synthesis of pure phase iron oxide NPs. These bands of Fe_2_O_3_ molecules vibration were also well reported by previous studies [42,43]. FTIR spectrum of IONPs reveals that natural compounds in the form of functional group attach with NPs due to electrostatic force present in Fe^+2^ metal ions. These interfaces suggest NPs as an ideal candidate to boost plant regeneration and development [44].

The optical properties of NPs are of immense importance in evaluating biological applications. It is imperative to know accurate information about optical features of nanoparticles as these characteristics evaluate their light visible potential at room temperature. Fig 3(c) depicts the UV-visible absorption spectra of the α-Fe_2_O_3_ solution. The colloidal suspension was visually observed from light reddish-brown to dark reddish-brown and absorption spectra were recorded in the range between 200-800 nm. The absorption peak at 200 nm shows α-Fe_2_O_3_-NPs formation of a single-phase rhombohedral structure as these peaks begin from the electronic transition between O and Fe. Bandgap energies of IONPs were calibrated by UV data and utilizing Tauc relation for direct bandgap material (αhν)^2^= A(hν - E_g_).

Where hν stands for photon energy, E_g_ represents bandgap energy, A is constant and α is the absorption coefficient. The linear examination of the (αhν)^2^ curves up to the energy axis show bandgap energy of IONPs. The E_g_ value of green synthesized IONPs is found to be 2.77 eV which is apparent from the earlier study [45].

The UV absorption phenomenon is due to surface plasmon resonance which may be result in a red shift or blue shift depending on the morphology of NPs, size, surface charge value, chemistry of solvent, and concentration in the solvent [46]. Surface activation of IONPs is linked to the polarization of metal complex that transferred the resonance in the optical region. The coarse surface of synthesized IONPs is due to the presence of plant natural compounds that are acting as surface capping agents. When NPs are synthesized via a green method, such coarseness in the morphology of NPs is common phenomenon [47]. However, it has been noted that irregular morphology of NPs can enhance their surface area which increases reactivity and boosts their activity in plant regeneration

The reactivity of NPs makes them differ from bulk material relates to the squeezing of an electron at the nanoscale which results in quantum confinement effect. This phenomenon can be studied by understanding change in bandgap energy, electron density, and number of atoms forming NPs [48]. Thus, we conclude from these findings that the overall reactivity of NPs depend on free electron density in the conduction band and the total number of atoms within NPs.

TGA analysis demonstrates the thermal behavior as well as capping action of the natural compounds from *C. jwarancusa* leaves extract on IONPs (Fig 3d). The amount of IONPs weight over time as temperature changes were examined in three phases. In the first phase, (100-200ºC) mass of NPs fluctuates due to surface adsorbed water loss. The main loss observed around 250-300ºC and 400-500ºC in the second phase is expectedly due to mass decomposition of chelating biomolecules (mainly phenolics and flavonoids) which are acting as surface stabilizing agents around IONPs. This evaluation is supported by ATR-FTIR of IONPs, which depicts the presence of a functional group of different natural compounds (Fig 3b). The combustion of natural compounds illustrates the successful capping of synthesized IONPs. The rapid oxygen loss was observed during third phase due to extreme high temperatures 700-800ºC [49].

**Fig 3.** (a) X-ray powder diffraction (XRD) patterns of biosynthesized α-Fe_2_O_3_ demonstrating Braggs diffraction peak associated to Fe crystallites planes, (b) ATR-FTIR spectrum of green synthesized IONPs showing bond vibrations coming from surface attached bioactive compounds and Fe-O, (c) UV-Vis spectrum of IONPs, and (d) Thermogravimetric Analysis (TGA) of the prepared IONPs.

**Effect of α-Fe_2_O_3_-NPs on callus induction frequency**

In the present study, effect of biosynthesized IONPs from *C. jwarancusa* leaves extract was investigated for its efficacy in tissue culture of chickpea. This is the first comprehensive study of IONPs on chickpea tissue culture. Fe play a significant role in different physiological processes including redox reaction, respiration, and chlorophyll biosynthesis [50]. Iron is the most important nutrient for plant metabolism and growth [51] and its deficiency is a common nutritional disorder in different crops, resulting in low yield and productivity. To examine callus induction frequency of chickpea, both varieties Punjab-Noor 09 and Bittle-98 explants, embryo axes (EA) and embryo axes plus cotyledon (EXC) were investigated on simple MS media without 2,4-D and IONPs. It was examined that both varieties were not capable to induce callus on simple MS medium. Subsequently, the roles of different concentrations of IONPs (1, 5, 10, 15, 20 mg/L) on callus induction of both chickpea varieties were investigated and data is documented in Table 1. Punjab Noor-09 cultivar of chickpea has shown highest callogenesis frequency among both varieties and its EXC explants showed intensively stimulated callus size at 15 mg/L with 96% callus induction frequency followed by 89% at 10 mg/L and 77% at 5 mg/L compared with control 16%. Nevertheless, EA explants of Punjab Noor-09 cultivar showed callus induction frequency 85% at 15 mg/L and 76% at 10 mg/L (Fig 4). Among both tested explants (EXC and EA) of Bittle-98 cultivar of chickpea, the highest callus induction was observed by EXC explants at 15 mg/L of IONPs with 90% increase in callus frequency. Moreover, EA (embryo axes) explants of Bittle-98, showed 78% callogenesis at 15 mg/L and 69% at 10 mg/L concentration while control callus exhibit 34% growth frequency. These findings suggested that IONPs are non-toxic to plants at optimum concentrations and increase the plant callus induction frequency. The increase in callus induction demonstrate that Fe as micronutrient can contribute to plant growth and development [25]. It has been observed that cellular metabolism as a regular function produces ROS which includes hydroxyl radicals, hydrogen peroxide, and superoxide anions. The cell keeps an exquisite balance of ROS removal and production to prevent cellular oxidative stress [52]. Different oxidation states of iron (Fe^+3^, Fe^+2^) act as ROS scavenger [29], preventing ROS induce mitochondrial destruction and DNA damage inside the cell and eventually increase chickpea growth dynamics.

Metallic nanoparticles have shown significant effect on plant growth and regeneration capacity [15] Nevertheless, we observed a reduction in callus induction frequency in both explants EA (31%) and EXC (44%) in Bittle-98 cultivar, when the concentration of IONPs were increased up to 20 mg/L level. A similar callus inhibition dynamics were observed at 20 mg/L concentration in EA (35%) and EXC (47%) explants of Punjab-Noor 09 cultivar. We conclude that the effect of IONPs was concentration-dependent and an increase in callogenesis was observed between 10-15 mg/L. Further increase in the concentration resulted in the negative response of IONPs which may be due to cell wall injury [53]. It is now well reported that higher concentrations of NPs show toxicity in both animals and plant tissues [54]. The IONPs at high concentration can restrict the electron transport chain of chloroplast and mitochondria, which may lead to oxidative burst with high ROS concentration, causing cell death [55], which in turn reduces the callus induction frequency.

Both variety of explants have shown different response in terms of optimum callus induction frequency at various concentrations of NPs. In Punjab-Noor 09 cultivar, both explants show more callogenesis frequency as compare to Bittle-98 cultivar. Hence, it can be suggested that nanomaterials having extremely small size may enter the explants and subsequently affect genetic reprogramming traits [56]. Likewise, effect of NPs in plants also depends on particles concentration, plant species, and exposure time [57].

In order to determine the effect of IONPs on organogenesis, calli were shifted to regeneration media supplemented with various concentrations of IONPs ranging from 1, 5, 10, 15 to 20 mg/L. Following transfer to regenerated media, calli show a stable increase in size on IONPs below 15 mg/L concentration in both types of media but display no organogenesis or increase in growth on other tested concentrations. Since calluses started to increase the size for a longer period (8 weeks) and NPs uptake increased biomass of callus cells, thus, these results encourage us to further study the direct shoot regeneration in chickpeas.

**Fig 4.** Effect of IONPs (1, 5, 10, 15, 20 mg/L) on callogenesis of chickpea varieties; EA, embryo axes with removed root apex, EXC embryo axes plus attached part of the cotyledon.

**Table 1**. Callus induction frequency of the chickpea cultivars on MS medium at different concentrations of IONPs.

|  | **Bittle-98 variety** | | **Punjab Noor-2009 variety** | |
| --- | --- | --- | --- | --- |
| **Treatments** | **EA** | **EXC** | **EA** | **EXC** |
| **Control (MS+2,4D)** | 20.5±1.1^f^ (34%) | 15.2±0.3^g^ (25%) | 23.6±0.2^e^ (39%) | 9.8±1.5^g^ (16%) |
| **IONPs (1 mg/L)** | 25.2±1.3^e^ (42%) | 36.7±1.1^d^ (61%) | 30.8±0.7^d^ (51%) | 42.2±0.1^c^ (70%) |
| **IONPs (5 mg/L)** | 34.0± 0.9^d^ (56%) | 42.1±0.6^c^ (70%) | 38.2±2.1^c^ (63%) | 46.2±0.8^b^ (77%) |
| **IONPs (10 mg/L)** | 41.5±1.5^c^ (69%) | 49.4±1.2^b^ (82%) | 45.8±1.2^b^ (76%) | 53.5±2.1^ab^ (89%) |
| **1ONPs (15 mg/L)** | 46.9±0.7^b^ (78%) | 54.1±2.3^a^ (90%) | 51.0±1.9^ab^ (85%) | 57.6±1.7^a^ (96%) |
| **IONPs (20 mg/L)** | 18.6±0.2^f^ (31%) | 26.4±1.6^e^ (44%) | 21.0±1.3^f^ (35%) | 28.4±0.9^d^ (47%) |

Values show the mean ± standard error (SE) of 20 replicates of each treatment. Values followed by small alphabets stands for the individual values as an average of three replicates (*P* < 0.05). Variable groups (explants, varieties and treatments) that are not represented by same letter are significantly different at (*P* < 0.05). EA, embryo axes with removed root apex, EXC embryo axes plus attached part of cotyledon.

**Effect of α-Fe_2_O_3_-NPs on organogenesis**

For direct shoot regeneration of chickpea, EXC and EA explants of both varieties were examined on simple MS medium without IONPs and growth hormones. We observed that both explants were unable to show any regeneration, like callus induction. Conversely, shoot regeneration capacity of explants on best-selected regeneration media (SRM I) was enhanced with the addition of different concentrations of IONPs (Fig 5). Like callogenesis, direct shoot regeneration of EXC explants showed highest frequency of regeneration 88% at 15 mg/L, compared with remaining tested concentration 10 mg/L (84%), 5 mg/L (78%) and 1 mg/L (59%) in Punjab-noor 2009 variety. These results indicate that by increasing the concentration of IONPs, shoot regeneration efficacy is also increased however, at high concentration (20 mg/L) shoot regeneration frequency was decreased to 48%. Similar results are reported by Shankramma et al. [26] in tomato plants which showed reduction in regeneration frequency at high concentration of iron oxide nanoparticles. When EXC explants of Bittle-98 variety were tested against the different concentration of IONPs, they demonstrated similar dose dependent response, 76% shoot regeneration efficiency at 15 mg/L, 69% at 10 mg/L, 60% at 5 mg/L and 46 at 1 mg/L. High frequency of direct shoot regeneration was observed in both variety explants, but best results were achieved at 15 mg/L concentration of IONPs. Iron play a significant role in metabolism such as plant respiration, photosynthesis, electron transfer in a redox reaction, and biosynthesis of chlorophyll and phytohormones [58]. It can therefore be assume that IONPs interact with plant metabolism and they may also adhere to the roots of plants and cause morphological and physiological changes [59].

The possible mechanism of action of NPs on plant cell is that NPs are extremely reactive, therefore, can easily be attached to the plant cell surface and Fe^+3^ released on the cell surface is easily absorbed into cells due to smaller particle size resulting in improved plant growth and development. The overall mechanism of IONPs as nano-fertilizer is proposed in Fig 6. Like callus induction, both explants in Punjab noor-2009 variety showed more regeneration frequency as compare to Bittle-98. The difference in shoot regeneration capability was observed among them (Table 2), however, compared with control, both varieties showed exceptional regeneration response. This shows that nanoparticle activity is directly related to the growth prospective of genotypes [60].

**Fig 5.** Effect of IONPs (1, 5, 10, and 15 mg/L) on organogenesis of chickpea varieties; EA, embryo axes EXC. Embryo axes plus attached part of cotyledon

**Fig 6.** The hypothesized mechanism of action of Hematite IONPs as nano-fertilizer to enhance regeneration capability of recalcitrant crops.

**Table 2**. Effect of different concentrations of IONPs on direct shoot regeneration frequency of chickpea varieties.

|  | **Bittle-98 variety** | | **Punjab Noor-2009 variety** | |
| --- | --- | --- | --- | --- |
| **Treatments** | **EA** | **EXC** | **EA** | **EXC** |
| **Control (MS+BAP)** | 12.6±1.5^g^ (21%) | 19.2±2.4^f^ (32%) | 21.1±1.3^e^ (35%) | 23.5±0.5^e^ (39%) |
| **IONPs (1 mg/L)** | 32.1± 0.7^d^ (53%) | 27.6±0.9^e^ (46%) | 33.0±0.5^d^ (55%) | 35.5±0.9^d^ (59%) |
| **IONPs (5 mg/L)** | 39.2±1.1^c^ (65%) | 36.0±1.5^d^ (60%) | 42.1±1.0^b^ (70%) | 46.8±2.6^ab^ (78%) |
| **IONPs (10 m g/L)** | 42.6±0.0^b^ (71%) | 41.5±1.8^b^ (69%) | 45.0±2.2^ab^ (75%) | 50.5±0.1^a^ (84%) |
| **1ONPs (15 mg/L)** | 47.4.0±0.4^a^ (79%) | 45.7±2.5^ab^ (76%) | 51.6±0.9^a^ (86%) | 53.0±1.5^a^ (88%) |
| **IONPs (20 mg/L)** | 24.1±2.1^e^ (40%) | 00.0±0.0 (0%) | 27.1±1.8^e^ (45%) | 29.0±1.8^d^ (48 %) |

Data followed by small alphabets stand for the individual as an average of three replicates (*P* < 0.05). Each replicate consists of 20 treatments. EA, embryo axes with removed root apex, EXC embryo axes plus attached part of the cotyledon.

**Effect of α-Fe_2_O_3_-NPs on shoot elongation and root induction**

About 2.2 cm shoot pieces were separated carefully from both variety explants and shifted to shoot elongation (SE) media with different concentrations of IONPs to observe shoot elongation (Table 3). The shoot length of each explant was recorded after 14 days of incubation. Our results indicated that EXC explants of Punjab-noor 09 variety induce highest shoot elongation followed by EA explants of the same variety. Mainly, the healthy and branched shoots produce on 15 mg/L concentration of IONPs showed a maximum length of 11.8±0.5 cm and 9.9±0.3 cm respectively (Fig 7a).

Rooting is considered as one of the key obstacles for efficient regeneration of chickpea. Scientists have developed different strategies to overcome this difficulty. Many of them utilize micrografting techniques, some researchers manipulated the rooting media (decrease salt concentrations), and others used pulse treatment with liquid media having auxins [61]. Although these methods increase root efficiency in chickpea, nonetheless, they are difficult to handle and are time consuming. A reliable chickpea regeneration system has not been available yet, because of the fact that it owns recalcitrant nature to regeneration [62]. Iron is used as an enzyme cofactor and being an indispensable element for the photosynthetic process. In recent years, iron based nanomaterial are used as fertilizers to enhance crop yield [63]. However, to the best of our knowledge, no attention has been paid earlier to examine the effect of IONPs on the type of explants utilizing to initiate the regeneration process on root induction. We tried our level best to study this aspect by taking regenerated shoots from each type of explants (EA and EXC) and assessed their rooting frequency. Results revealed that embryo axes with adjacent cotyledon explants (EXC) show higher rooting pattern than shoot regenerated from other type embryo axes (EA) explants. Similar finding is also reported by Amer et al. [61] suggesting that type of explants has a direct effect on shoots and root regeneration ability. After 10 days of incubation of regenerated shoots with different concentrations of IONPs (1-15 mg/L), highest root induction was observed in EXC explants of Punjab-Noor variety; 83% root induction at 15 mg/L concentration, 71% at 10 mg/L, 62% at 5 mg/L, while 32% root induction was observed in control plants (Table 3). EA explants of Punjab Noor-09 variety show 75% root induction at 15 mg/L. In While Bittle 98 variety, both explants show 78% (EXC) and 69% (EA) root induction at 15 mg/L concentration. The maximum rooting response with healthy roots and root hairs were observed at a concentration of 15 mg/L followed by 10 mg/L. Phenotypic analysis of roots revealed that IONPs (1, 5, 10, 15 mg/L) treated explants displayed a concentration-dependent response in root length and branching. Our results proved that different concentrations of IONPs are highly effective for root induction in chickpea plants and this study is a contribution towards improving regeneration dynamics and transformation system of chickpea particularly and legumes in general.

**Table 3.** Effect of different concentrations of IONPs on root induction frequency of chickpea varieties.

|  | **Bittle-98 variety** | | **Punjab Noor-2009 variety** | |
| --- | --- | --- | --- | --- |
| **Treatments** | **EA** | **EXC** | **EA** | **EXC** |
| **Control (MS+IBA)** | 11.0±0.8^a^ (18%) | 13.2±0.5^ab^ (22%) | 10.2±0.0^a^ (16%) | 19.3±0.2^ab^ (32%) |
| **IONPs (1 mg/L)** | 21.6± 0.6^ab^ (36%) | 29.5±2.1^b^ (49%) | 24.7±1.7^b^ (41%) | 33.1±1.2^c^ (55%) |
| **IONPs (5 mg/L)** | 28.2±2.1^b^ (47%) | 36.0±1.6^c^ (60%) | 31.4±1.5^bc^ (52%) | 37.2±1.0^c^ (62%) |
| **IONPs (10 mg/L)** | 35.0±0.7^c^ (58%) | 40.2±1.8^cd^ (67) | 38.5±0.8^cd^ (64%) | 42.6±1.3^d^ (71%) |
| **1ONPs (15 mg/L)** | 41.5±1.5^cd^ (69%) | 47.0±2.6^e^ (78%) | 45.0±1.2^d^ (75%) | 49.8±0.9^e^ (83%) |

Values show the mean ± standard error (SE) of 20 replicates each treatment. Values followed by small alphabets stand for the individual values as an average of three replicates (*P* < 0.05). Variable groups (explants, varieties and treatments) that are not represented by the same letter are significantly different (*P* < 0.05). EA, embryo axes with removed root apex, EXC embryo axes plus attached part of cotyledon

**Iron content in the regenerated chickpeas**

Iron content in regenerated chickpea plantlets were quantified by inductively coupled plasma-optical emission spectrometry. As shown in Fig 7b, iron content per mg dry weight of regenerated shoots cultured for 21 days under white fluorescence light with different concentrations of IONPs (1-20 mg L^-1^). Control regenerated shoots contained 1.89 mg of Fe^+2^ g^-1^ dry weight, while IONPs treated explants showed significantly higher iron content in chickpea regenerated plantlets. Results demonstrate that in all experimental group, IONPs were translocated in all shoots. However, Punjab noor-09 EXC explant shoots cultured at 15 mg L^-1^ concentration of IONPs contain significant amount of iron 4.21 mg g^-1^ dry weight. These findings are in line with previous results obtained from callogenesis (Fig 4) and shoot regeneration (Fig 5). It seems that hematite iron oxide nanoparticles with 50 nm size, contributed well to enhancing regeneration and growth in chickpea explants by providing iron ions. Iron is an important micronutrient involves in a different physiological reaction and is an imperative component of chlorophyll [64]. Our results also reveal that iron concentration has increased in chickpea shoots with increasing concentrations of NPs. This could be due to the biomineralization and internalization of localized iron in different parts of the chickpea plant. The results suggest that smaller particle size can dissolve iron ions more effectively in plantlets and more ions are provided to chickpea regenerated plants, resulting in rapid plant growth. Thus, iron can be used as a nano-fertilizer to enhance plant yield and productivity.

**Fig 7.** (a) Effect of different concentrations of IONPs on shoot length of proliferating shoots from different explants; EXC and EA, and (b) Iron content per dry weight of regenerated chickpea plantlets.

**Conclusion**

Green synthesis has proved to be a more biocompatible, dependable, and economical method to fabricate extremely small-sized metallic oxide nanoparticles. In the current study, iron oxide nanoparticles have efficaciously synthesized from *C. jwarancusa* leaves by a novel green engineering approach. Iron (III) nitrate was used as a precursor while *C. jwarancusa* aqueous extract was used as a capping and stabilizing agent. The results show that green synthesized hematite NPs are rod-shaped with a size of 50±0.2 nm while XRD confirms the highly crystalline nature of α-Fe_2_O_3_ with higher reactivity. ATR-FTIR confirms the presence of Fe-O stretch. IONPs as nano fertilizer were effectively supplemented to chickpea explants and enhanced the growth pattern. We found that an optimum dose of IONPs is adequate to promote callogenesis and regeneration dynamics in chickpea plantlets. The regeneration potential of chickpea gives very promising results and provides a dual effect. This will keep plant seedlings infection-free and provide an excellent environment for rapid regeneration and growth. Keeping in view the importance of iron oxide as nano-fertilizer, it also provides a complete package to the farmers for higher economical yield of chickpea. Overall, this study shows an increase in growth parameters of chickpea plant using IONPs and we reported no toxic effect of NPs on plant growth. These observations encourage that IONPs could be used green nano-nutrition in agronomic crops for environmental sustainability. Nevertheless, further studies at molecular level are needed for further considerations and the use of IONPs for more crop productivity and abiotic and biotic stress resistance.

**Competing of interest**

The authors have declared that no competing interests exist.

**Author Contributions**

Conceptualization, S.I., N.J., K.S.A., S.S., T.F.K, H.G., S.A, NIS, and S.Z.H; writing-original draft preparation, S.I., TFK., HG; writing- review and editing N.J., K.S.A., S.S., S.A., S.Z.H., AM. All authors have read and agreed to publish version of the manuscript.

**References**

1. Food and agriculture organization of the United Nations- 2019- World food and agriculture-statistical pocketboo.pdf. http://www.fao.org/3/ca6463en/ca6463en.pdf

2. Nedumaran S, Abinaya P, Jyosthnaa P, Shraavya B, Rao P, Bantilan C. Consumption and Trade Trends in Developing Countries. 2015; 67.

3. Merga B, Haji J. Economic importance of chickpea: Production, value, and world trade. Yildiz F, editor. Cogent Food & Agriculture. 2019; 5(1):1615718 doi:10.1080/23311932.2019.1615718

4. Jukanti AK, Gaur PM, Gowda CLL, Chibbar RN. Nutritional quality and health benefits of chickpea (Cicer arietinum L.): a review. Br J Nutr. 2012; 108 Suppl 1: S11-26. doi:10.1017/S0007114512000797

5. Varshney RK, Mohan SM, Gaur PM, Chamarthi SK, Singh VK, Srinivasan S, et al. Marker-Assisted Backcrossing to Introgress Resistance to Fusarium Wilt Race 1 and Ascochyta Blight in C 214, an Elite Cultivar of Chickpea. The Plant Genome. 2014;7: plantgenome. 2013.10.0035. doi:10.3835/plantgenome2013.10.0035

6. Ngwe T, Nukui Y, Oyaizu S, Takamoto G, Koike S, Ueda K, et al. Bean husks as a supplemental fiber for ruminants: potential use for activation of fibrolytic rumen bacteria to improve main forage digestion. Anim Sci J; 2012;83(1):43-9. sdoi:10.1111/j.1740-0929.2011.00916.x

7. Das Bhowmik SS, Cheng AY, Long H, Tan GZH, Hoang TML, Karbaschi MR, et al. Robust Genetic Transformation System to Obtain Non-chimeric Transgenic Chickpea. Front Plant Sci. 2019;10: 524. doi:10.3389/fpls.2019.00524

8. Thudi M, Chitikineni A, Liu X, He W, Roorkiwal M, Yang W, et al. Recent breeding programs enhanced genetic diversity in both desi and kabuli varieties of chickpea (Cicer arietinum L.). Sci Rep. 2016; 6: 38636. doi:10.1038/srep38636

9. Gurjar GS, Giri AP, Gupta VS. Gene Expression Profiling during Wilting in Chickpea Caused by &lt;i&gt;Fusarium oxysporum&lt;/i&gt; F. sp. &lt;i&gt;Ciceri&lt;/i&gt; AJPS. 2012;03: 190–201. doi:10.4236/ajps.2012.32023

10. Atif RM, Patat-Ochatt EM, Svabova L, Ondrej V, Klenoticova H, Jacas L, et al. Gene Transfer in Legumes. In: Lüttge U, Beyschlag W, Francis D, Cushman J, editors. Progress in Botany: Vol 74. Berlin, Heidelberg: Springer; 2013; pp. 37–100. doi:10.1007/978-3-642-30967-0_2

11. Alharby H, Metwali E, Fuller M, Aldhebiani A. Impact of application of zinc oxide nanoparticles on callus induction, plant regeneration, element content and antioxidant enzyme activity in tomato (Solanum lycopersicum Mill.) under salt stress. Archives of Biological Sciences. 2016;68: 723–735. doi:10.2298/ABS151105017A

12. Mahna N. Plant In vitro Culture goes Nano: Nanosilver-Mediated Decontamination of Ex vitro Explants. J Nanomedic Nanotechnol. 2013;04. doi:10.4172/2157-7439.1000161

13. Chen H. Metal based nanoparticles in agricultural system: behavior, transport, and interaction with plants. Chemical Speciation & Bioavailability. 2018;30: 123–134. doi:10.1080/09542299.2018.1520050

14. Sharma JK, Srivastava P, Akhtar MS, Singh G, Ameen S. α-Fe2O3 hexagonal cones synthesized from the leaf extract of Azadirachta indica and its thermal catalytic activity. New J Chem. 2015;39: 7105–7111. doi:10.1039/C5NJ01344E

15. Nalci OB, Nadaroglu H, Pour AH, Gungor AA, Haliloglu K. Effects of ZnO, CuO and γ-Fe3O4 nanoparticles on mature embryo culture of wheat (Triticum aestivum L.). Plant Cell Tiss Organ Cult. 2019;136: 269–277. doi:10.1007/s11240-018-1512-8

16. Pereira A do ES, Oliveira HC, Fraceto LF. Polymeric nanoparticles as an alternative for application of gibberellic acid in sustainable agriculture: a field study. Sci Rep. 2019;9: 7135. doi:10.1038/s41598-019-43494-y

17. Pacheco I, Buzea C. Nanoparticle Uptake by Plants: Beneficial or Detrimental? In: Faisal M, Saquib Q, Alatar AA, Al-Khedhairy AA, editors. Phytotoxicity of Nanoparticles. Cham: Springer International Publishing; 2018. pp. 1–61. doi:10.1007/978-3-319-76708-6_1

18. Karny A, Zinger A, Kajal A, Shainsky-Roitman J, Schroeder A. Therapeutic nanoparticles penetrate leaves and deliver nutrients to agricultural crops. Scientific Reports. 2018;8: 7589. doi:10.1038/s41598-018-25197-y

19. Abusalem M, Awwad A, Ayad J, Rayyan AA. Green Synthesis of α-Fe2O3 Nanoparticles Using Pistachio Leaf Extract Influenced Seed Germination and Seedling Growth of Tomatos. 2019; 6.

20. Marcus M, Karni M, Baranes K, Levy I, Alon N, Margel S, et al. Iron oxide nanoparticles for neuronal cell applications: uptake study and magnetic manipulations. Journal of Nanobiotechnology. 2016;14: 37. doi:10.1186/s12951-016-0190-0

21. Atyam KK, Ghosh A, Mukherjee K, Majumder SB. Hematite iron oxide nano-particles: facile synthesis and their chemi-resistive response towards hydrogen. Mater Res Express. 2015;2: 055901. doi:10.1088/2053-1591/2/5/055901

22. Asoufi HM, Al-Antary TM, Awwad AM. Green route for synthesis hematite (α-Fe 2 O 3 ) nanoparticles: Toxicity effect on the green peach aphid, Myzus persicae (Sulzer). Environ Nanotechnol Monit Manag. 2018;9:107–111. doi:10.1016/j.enmm.2018.01.004

23. Gupta AK, Gupta M. Synthesis and surface engineering of iron oxide nanoparticles for biomedical applications. Biomaterials. 2005;26:3995–4021. doi:10.1016/j.biomaterials.2004.10.012

24. Mozafari A akbar, Havas F, Ghaderi N. Application of iron nanoparticles and salicylic acid in in vitro culture of strawberries (*Fragaria ananassa* Duch.) to cope with drought stress. Plant cell, tissue, and organ culture. 2018.

25. Li J, Hu J, Ma C, Wang Y, Wu C, Huang J, et al. Uptake, translocation and physiological effects of magnetic iron oxide (γ-Fe2O3) nanoparticles in corn (*Zea mays* L.). Chemosphere. 2016;159: 326–334. doi:10.1016/j.chemosphere.2016.05.083

26. Shankramma K, Yallappa S, Shivanna MB, Manjanna J. Fe2O3 magnetic nanoparticles to enhance *S. lycopersicum* (tomato) plant growth and their biomineralization. Appl Nanosci. 2016;6: 983–990. doi:10.1007/s13204-015-0510-y

27. Maswada HF, Djanaguiraman M, Prasad PVV. Seed treatment with nano-iron (III) oxide enhances germination, seeding growth and salinity tolerance of sorghum. J Agron Crop Sci. 2018;204: 577–587. doi:10.1111/jac.12280

28. Al-Amri N, Tombuloglu H, Slimani Y, Akhtar S, Barghouthi M, Almessiere M, et al. Size effect of iron (III) oxide nanomaterials on the growth, and their uptake and translocation in common wheat (*Triticum aestivum* L.). Ecotoxicol Environ Safe. 2020;194: 110377. doi:10.1016/j.ecoenv.2020.110377

29. Roschzttardtz H, Conéjéro G, Divol F, Alcon C, Verdeil J-L, Curie C, et al. New insights into Fe localization in plant tissues. Front Plant Sci. 2013;4. doi:10.3389/fpls.2013.00350

30. Irum S, Tabassum S, Qureshi R, Gulfraz M, Anwar P. Phytochemical analysis of medicinally important constituents of *Teucrium stocksianum* Boiss. Pak J Bot. 2019;51. doi:10.30848/PJB2019-3(17)

31. Khalil AT, Ovais M, Ullah I, Ali M, Shinwari ZK, Maaza M. Biosynthesis of iron oxide (Fe_2_O_3_) nanoparticles via aqueous extracts of *Sageretia thea* (Osbeck.) and their pharmacognostic properties. Green Chem Lett Rev. 2017;10: 186–201. doi:10.1080/17518253.2017.1339831

32. Manickavasagam M, Pavan G, Vasudevan V. A comprehensive study of the hormetic influence of biosynthesized AgNPs on regenerating rice calli of indica cv. IR64. Sci Rep. 2019;9: 8821. doi:10.1038/s41598-019-45214-y

33. Jahani S, Saadatmand S, Mahmoodzadeh H, Khavari-Nejad RA. Effects of cerium oxide nanoparticles on biochemical and oxidative parameters in marigold leaves. Toxicol Environ Chem. 2018;100: 677–692. doi:10.1080/02772248.2019.1587443

34. Al-Ruqeishi MS, Mohiuddin T, Al-Saadi LK. Green synthesis of iron oxide nanorods from deciduous Omani mango tree leaves for heavy oil viscosity treatment. Arabian Journal of Chemistry. 2019;12: 4084–4090. doi:10.1016/j.arabjc.2016.04.003

35. Aisida S, Madubuonu N, Alnasir M, Ahmad I, Botha S, Maaza M, et al. Biogenic synthesis of iron oxide nanorods using Moringa oleifera leaf extract for antibacterial applications. Appl Nanosci. 2019. doi:10.1007/s13204-019-01099-x

36. Fatemi M, Mollania N, Momeni-Moghaddam M, Sadeghifar F. Extracellular biosynthesis of magnetic iron oxide nanoparticles by Bacillus cereus strain HMH1: Characterization and in vitro cytotoxicity analysis on MCF-7 and 3T3 cell lines. J Biotechnol. 2018;270: 1–11. doi:10.1016/j.jbiotec.2018.01.021

37. Jacob J, Khadar M. VSM and Mössbauer study of nanostructured hematite. Journal of Magnetism and Magnetic Materials. J Magn Magn Mater. 2010;322: 614–621. doi:10.1016/j.jmmm.2009.10.025

38. Trpkov D, Panjan M, Kopanja L, Tadic M. Hydrothermal synthesis, morphology, magnetic properties and self-assembly of hierarchical α-Fe 2 O 3 (hematite) mushroom-, cube- and sphere-like superstructures. Appl Surf Sci. 2018;457. doi:10.1016/j.apsusc.2018.06.224

39. Lassoued A, Dkhil B, Gadri A, Ammar S. Control of the shape and size of iron oxide (α-Fe2O3) nanoparticles synthesized through the chemical precipitation method. Results Phys. 2017;7: 3007–3015. doi:10.1016/j.rinp.2017.07.066

40. Wahab R, Khan F, Al-Khedhairy AA. Hematite iron oxide nanoparticles: apoptosis of myoblast cancer cells and their arithmetical assessment. Rsc Adv. 2018;8: 24750–24759. doi:10.1039/C8RA02613K

41. Jahangirian H (kamran), Rafiee Moghaddam R, Jahangirian N, Nikpey B, Jahangirian S, Bassous N, et al. Green Synthesis of Zeolite/Fe2O3 Nanocomposites: Toxicity & Cell Proliferation Assays and Application as a Smart Iron Nanofertilizer. Int J Nanomedicine. 2020; 15: 1005–1020. doi:10.2147/IJN.S231679

42. Ebrahiminezhad A, Zare-Hoseinabadi A, Berenjian A, Younes G. Green synthesis and characterization of zero-valent iron nanoparticles using stinging nettle (Urtica dioica) leaf extract. Green Processing Syn. 2017;6. doi:10.1515/gps-2016-0133

43. Jadidi Kouhbanani MA, Beheshtkhoo N, Amani AM, Taghizadeh S, Beigi V, Zakeri Bazmandeh A, et al. Green synthesis of iron oxide nanoparticles using Artemisia vulgaris leaf extract and their application as a heterogeneous Fenton-like catalyst for the degradation of methyl orange. Mater Res Express. 2018;5: 115013. doi:10.1088/2053-1591/aadde8

44. Servin A, Elmer W, Mukherjee A, De la Torre-Roche R, Hamdi H, White JC, et al. A review of the use of engineered nanomaterials to suppress plant disease and enhance crop yield. J Nanopart Res. 2015;17: 92. doi:10.1007/s11051-015-2907-7

45. Farahmandjou M, Soflaee F. Low Temperature Synthesis of α-Fe2O3 Nano-rods Using Simple Chemical Route. J Nanostruct. 2014; 7. 413-418.

46. Naz S, Islam M, Tabassum S, Fernandes NF, Carcache de Blanco EJ, Zia M. Green synthesis of hematite (α-Fe2O3) nanoparticles using Rhus punjabensis extract and their biomedical prospect in pathogenic diseases and cancer. J Mol Struct. 2019;1185: 1–7. doi:10.1016/j.molstruc.2019.02.088

47. Gm S, At T, As N. Biosynthesis, Characterization of Magnetic Iron Oxide Nanoparticles and Evaluations of the Cytotoxicity and DNA Damage of Human Breast Carcinoma Cell Lines. In: Artificial cells, nanomedicine, and biotechnology. Artif Cells Nanomed Biotechnol; 2018. doi:10.1080/21691401.2017.1366335

48. Gharibshahi L, Saion E, Gharibshahi E, Shaari AH, Matori KA. Structural and Optical Properties of Ag Nanoparticles Synthesized by Thermal Treatment Method. Materials (Basel). 2017;10. doi:10.3390/ma10040402

49. Lohrasbi S, Kouhbanani MAJ, Beheshtkhoo N, Ghasemi Y, Amani AM, Taghizadeh S. Green Synthesis of Iron Nanoparticles Using Plantago major Leaf Extract and Their Application as a Catalyst for the Decolorization of Azo Dye. BioNanoSci. 2019;9: 317–322. doi:10.1007/s12668-019-0596-x

50. Zargar SM, Agrawal GK, Rakwal R, Fukao Y. Quantitative proteomics reveals role of sugar in decreasing photosynthetic activity due to Fe deficiency. Front Plant Sci. 2015;6. doi:10.3389/fpls.2015.00592

51. Samaranayake P, Peiris BD, Dssanayake S. Effect of Excessive Ferrous (Fe^2+^) on Growth and Iron Content in Rice (*Oryza sativa*). Int J Agri Biol. 14(2):296-298.

52. Ferreira CA, Ni D, Rosenkrans ZT, Cai W. Scavenging of reactive oxygen and nitrogen species with nanomaterials. Nano Res. 2018;11: 4955–4984. doi:10.1007/s12274-018-2092-y

53. Kokina I, Mickeviča I, Jahundoviča I, Ogurcovs A, Krasovska M, Jermaļonoka M, et al. Plant Explants Grown on Medium Supplemented with Fe _3_ O _4_ Nanoparticles Have a Significant Increase in Embryogenesis. J Nanomaterials. 2017;2017: 1–11. doi:10.1155/2017/4587147

54. Nguyen NHA, Padil VVT, Slaveykova VI, Černík M, Ševců A. Green Synthesis of Metal and Metal Oxide Nanoparticles and Their Effect on the Unicellular Alga Chlamydomonas reinhardtii. Nanoscale Res Lett. 2018;13: 159. doi:10.1186/s11671-018-2575-5

55. Khan MN, Mobin M, Abbas ZK, AlMutairi KA, Siddiqui ZH. Role of nanomaterials in plants under challenging environments. Plant Physiol Biochem. 2017;110: 194–209. doi:10.1016/j.plaphy.2016.05.038

56. Martínez-Fernández D, Barroso D, Komárek M. Root water transport of Helianthus annuus L. under iron oxide nanoparticle exposure. Environ Sci Pollut Res Int. 2016;23: 1732–1741. doi:10.1007/s11356-015-5423-5

57. Zuverza-Mena N, Martínez-Fernández D, Du W, Hernandez-Viezcas JA, Bonilla-Bird N, López-Moreno ML, et al. Exposure of engineered nanomaterials to plants: Insights into the physiological and biochemical responses-A review. Plant Physiol Biochem. 2017;110: 236–264. doi:10.1016/j.plaphy.2016.05.037

58. Sánchez-Alcalá I, del Campillo M del C, Barrón V, Torrent J. Evaluation of preflooding effects on iron extractability and phytoavailability in highly calcareous soil in containers. J Plant Nutri Soil Sci. 2014;177: 150–158. doi:10.1002/jpln.201200302

59. Tombuloglu H, Slimani Y, Alshammari T, Kekec G, Almessiere M, Baykal A, et al. Magnetic Behavior and Nutrient Content Analyses of Barley (Hordeum vulgare L.) Tissues upon CoNd0.2Fe1.8O4 Magnetic Nanoparticle Treatment. J Soil Sci Plant Nutri. 2019. doi:10.1007/s42729-019-00115-x

60. Van NL, Ma C, Shang J, Rui Y, Liu S, Xing B. Effects of CuO nanoparticles on insecticidal activity and phytotoxicity in conventional and transgenic cotton. Chemosphere. 2016;144: 661–670. doi:10.1016/j.chemosphere.2015.09.028

61. Amer A, Mohamed G, Pantaleo V, Leonetti P, Hanafy MS. *In vitro* regeneration through organogenesis in Egyptian chickpea. Plant Biosyst. 2019;153:835–842. doi:10.1080/11263504.2018.1549616

62. Kumari P, Singh S, Yadav S, Tran LSP. Pretreatment of seeds with thidiazuron delimits its negative effects on explants and promotes regeneration in chickpea (*Cicer arietinum* L.). Plant Cell Tiss Organ Cult. 2018;133: 103–114. doi:10.1007/s11240-017-1365-6

63. Palchoudhury S, Jungjohann KL, Weerasena L, Arabshahi A, Gharge U, Albattah A, et al. Enhanced legume root growth with pre-soaking in α-Fe2O3 nanoparticle fertilizer. RSC Adv. 2018;8: 24075–24083. doi:10.1039/C8RA04680H

64. Pariona N, Martínez AI, Hernandez-Flores H, Clark-Tapia R. Effect of magnetite nanoparticles on the germination and early growth of Quercus macdougallii. Sci Total Environ. 2017;575: 869–875. doi:10.1016/j.scitotenv.2016.09.128

65. Shafique S, Jabeen N, Ahmad KS, Irum S, Anwaar S, Ahmad N, Alam S, Ilyas M, Khan TF, Hussain SZ. Green fabricated zinc oxide nanoformulated media enhanced callus induction and regeneration dynamics of Panicum virgatum L. PLoS ONE 2020;15(7): e0230464. https://doi.org/10.1371/journal.pone.0230464
